# A multicenter, community-based, mixed methods assessment of the acceptability of a triple drug regimen for elimination of lymphatic filariasis

**DOI:** 10.1371/journal.pntd.0009002

**Published:** 2021-03-03

**Authors:** Alison Krentel, Nandha Basker, Madsen Beau de Rochars, Joshua Bogus, Daniel Dilliott, Abdel N. Direny, Christine Dubray, Peter U. Fischer, Adriani Lomi Ga, Charles W. Goss, Myra Hardy, Cade Howard, Purushothaman Jambulingam, Christopher L. King, Moses Laman, Jean Frantz Lemoine, Shruti Mallya, Leanne J. Robinson, Josaia Samuela, Ken B. Schechtman, Andrew C. Steer, Taniawati Supali, Livingstone Tavul, Gary J. Weil

**Affiliations:** 1 Bruyère Research Institute, Ottawa, Canada; 2 School of Epidemiology and Public Health, University of Ottawa, Ottawa, Canada; 3 ICMR-Vector Control Research Centre, Puducherry, India; 4 University of Florida, Ottawa, Florida, United States of America; 5 Washington University, St. Louis, Missouri, United States of America; 6 RTI Envision, Washington D.C., United States of America; 7 Centers for Disease Control and Prevention, Atlanta, Georgia, United States of America; 8 Government of East Nusa Tenggara, Kupang, Indonesia; 9 Murdoch Children’s Research Institute, Melbourne, Australia; 10 Case Western Reserve University, Cleveland, Ohio, United States of America; 11 Papua New Guinea Institute of Medical Research, Madang, Papua New Guinea; 12 Ministère de la Santé Publique et de la Population, Port au Prince, Haiti; 13 Burnet Institute, Melbourne, Australia; 14 Ministry of Health and Medical Services Fiji, Suva, Fiji; 15 Fiji Program Support Facility, Coffey Tetra Tech Company, Fiji; 16 Universitas Indonesia, Jakarta, Indonesia; University Hospital Bonn, GERMANY

## Abstract

**Background:**

Many countries will not reach elimination targets for lymphatic filariasis in 2020 using the two-drug treatment regimen (diethylcarbamazine citrate [DEC] and albendazole [DA]). A cluster-randomized, community-based safety study performed in Fiji, Haiti, India, Indonesia and Papua New Guinea tested the safety and efficacy of a new regimen of ivermectin, DEC and albendazole (IDA).

**Methodology/Principal findings:**

To assess acceptability of IDA and DA, a mixed methods study was embedded within this community-based safety study. The study objective was to assess the acceptability of IDA versus DA. Community surveys were performed in each country with randomly selected participants (>14 years) from the safety study participant list in both DA and IDA arms. In depth interviews (IDI) and focus group discussions (FGD) assessed acceptability-related themes. In 1919 individuals, distribution of sex, microfilariae (Mf) presence and circulating filarial antigenemia (CFA), adverse events (AE) and age were similar across arms. A composite acceptability score summed the values from nine indicators (range 9–36). The median (22.5) score indicated threshold of acceptability. There was no difference in scores for IDA and DA regimens. Mean acceptability scores across both treatment arms were: Fiji 33.7 (95% CI: 33.1–34.3); Papua New Guinea 32.9 (95% CI: 31.9–33.8); Indonesia 30.6 (95% CI: 29.8–31.3); Haiti 28.6 (95% CI: 27.8–29.4); India 26.8 (95% CI: 25.6–28) (P<0.001). AE, Mf or CFA were not associated with acceptability. Qualitative research (27 FGD; 42 IDI) highlighted professionalism and appreciation for AE support. No major concerns were detected about number of tablets. Increased uptake of LF treatment by individuals who had never complied with MDA was observed.

**Conclusions/Significance:**

IDA and DA regimens for LF elimination were highly and equally acceptable in individuals participating in the community-based safety study in Fiji, Haiti, India, Indonesia, and Papua New Guinea. Country variation in acceptability was significant. Acceptability of the professionalism of the treatment delivery was highlighted.

## Author summary

The acceptability of a new combination treatment regimen for lymphatic filariasis (ivermectin, plus DEC and albendazole, or IDA) was assessed as part of a larger community-based safety study in Fiji, Haiti, India, Indonesia and Papua New Guinea. To understand how trial participants and communities felt about the new treatment, a survey was carried out with people who had participated in the safety study receiving either the standard treatment [DEC plus albendazole (DA)] or the new treatment regimen (IDA). Focus group discussions and in-depth interviews were performed in the same communities. Results showed that there was no difference in acceptability between the DA and IDA. Adverse events and presence of filarial infection did not affect acceptability. The most important indicator associated with acceptability was country. All countries accepted the treatment regimens. Fiji had the highest acceptability scores, followed by Papua New Guinea, Indonesia, Haiti and India. Results from the qualitative research showed that study participants appreciated the professionalism of the drug delivery team and the support offered for the management of any adverse events.

## Introduction

Lymphatic filariasis (LF) is a neglected tropical disease (NTD) that is transmitted by nematode parasites [1]. Infection can cause long term clinical manifestations including hydrocele, lymphedema and elephantiasis [2,3]. The social and economic consequences of LF include disability, reduced earning potential, social exclusion, mental illness and costs associated with long-term medical care [4,5]. The World Health Organization has called for the elimination of LF as a public health problem by 2020 through two intervention pillars: 1) preventive chemotherapy for all eligible persons living in endemic areas and 2) morbidity management and disability prevention for those with clinically significant filarial disease [6,7].

Important achievements have been made since the launch of the Global Programme to Eliminate LF (GPELF) in 2000. More than eight billion treatments have been delivered to over 923 million people at least once through mass drug administration (MDA) [8]. Presently, 22 countries have stopped MDA for LF and are now conducting active surveillance to ensure that transmission of the disease-causing parasite has been interrupted [8]. Despite these impressive gains, many countries will not soon reach disease elimination targets using the current two-drug MDA regimens [diethylcarbamazine citrate (DEC) plus albendazole or ivermectin plus albendazole].

Clinical trials in Papua New Guinea (PNG) demonstrated that a triple drug regimen of ivermectin, DEC and albendazole (IDA) was more effective for clearing *Wuchereria bancrofti* microfilaremia than the standard two-drug DEC and albendazole regimen (DA) [9,10]. This IDA regimen was perceived as a potential way to accelerate LF elimination in some settings. A cluster-randomized, community-based safety study performed in five countries (Fiji, Haiti, India, Indonesia and PNG) showed that IDA was as safe as DA and that rates and types of adverse events (AE) were similar after either treatment [11]. The current paper reports results from a mixed methods acceptability study that was embedded within the five-country safety study.

Although acceptability studies have not been commonly performed in the context of NTD interventions, they are often used to aid or assess implementation of other types of health, educational and behavioral interventions [12–14]. The acceptability of a complex health intervention by both implementers and consumers has been identified as an important determinant of its feasibility [15]. Acceptability has been defined by Sekhon, Cartwright, & Francis (2017) as “a multi-faceted construct that reflects the extent to which people delivering or receiving a healthcare intervention consider it to be appropriate, based on anticipated or experienced cognitive and emotional responses to the intervention” [16]. As part of the guidelines review process at the World Health Organization, acceptability is considered as an important factor in the policy change process when new interventions are introduced.

Prior studies have identified several factors that contribute to the acceptability of a health intervention. Acceptable interventions should be effective, minimally restrictive, minimally intrusive and convenient [17–23]. The characteristics of a treatment used in clinical health interventions may also influence the acceptability of the intervention, including the treatment’s formulation, application, perceived benefits and the process used to deliver the treatment [24]. Interventions that produce fewer side effects and require less time or financial commitments from implementers or participants are generally considered more acceptable than those that do not [16,21,25,26]. Other factors that influence acceptability include whether the intervention aligns with the pre-existing value system of the society where it is to be implemented and whether consumers understand and feel capable of performing the behaviors required by the intervention [24,27,28]. Finally, acceptability can be influenced by the sociodemographic characteristics of the individuals comprising an intervention’s target population, including age, sex, geographic location, and the severity of the health problem being addressed [20,22,23,29,30].

Within the context of this research, the feasibility of introducing the new IDA regimen within LF-endemic communities is subject to its acceptability as compared to the standard DA regimen. This paper presents aggregated results from a five-country acceptability study that informed development of new guidelines for use of IDA in the global LF elimination program [31].

## Methods

### Ethics statement

Ethical approval for this study was received from: Bruyère Research Institute (M16-16-041) (Canada); Washington University at St. Louis, USA (ID: 201607068); Case Western Reserve University, Cleveland, USA (No. 10-16-08); Universitas Indonesia, Jakarta (No. 628/UN2.F1/Etik/VIII/2016); Papua New Guinea Medical Research Advisory (No 16.07); Ministère de la Santé et de la Population, Port-au-Prince, Haiti (ref 1415–66); Indian Council of Medical Research (IEC/IRB No. IHEC-0316/RJ); Human Research Ethics Committee, Royal Children’s Hospital Melbourne, Australia (Ref. 36205) and Fiji National Health Research and Ethics Review Committee, Suva, Fiji (2016.81.MC). The study is registered on clinicaltrials.gov: NCT02899936.

The study used a concurrent mixed methods research design combining a cross-sectional survey with focus group discussions (FGD) and in-depth interviews (IDI) with key informants. Qualitative and quantitative data were collected within four months of enrollment in the safety trial and within the same reference populations. (S1 Protocol)

### Development of study instruments

The survey was developed using questions derived from validated scales used to assess social validity, for which acceptability is a key component. Questions assessed factors relevant to compliance based on the literature [32,33]. Relevant questions from the ‘Treatment acceptability rating form-revised’ [34] as well as the ‘Intervention rating profile’ [35] were modified and incorporated into the survey. To assess acceptability with regards to product formulation and application, we addressed perceptions about taste and the number of pills, as well as feelings related to the number of pills, using a series of five faces expressing emotions through frowns, neutral faces and smiles [36]. The final questionnaire was subsequently assessed by co-investigators and tested in the field to ensure clarity and relevance to the local context. The questionnaire contained a total of 54 closed questions.

Topic guides for the FGDs explored the following themes: perceived health and social benefits of taking LF treatment; practice of directly observed therapy; perceptions about the number of pills; reasons why people take (do not take) LF treatment; adverse events (AE) experience; suggestions to promote MDA in the future in their communities.

Topic guides for the IDIs with key informants explored the following themes: perceived advantages of IDA versus DA; opportunities, concerns and potential challenges related to use of IDA; perceptions about the number of pills; perceptions and experiences regarding AE in their communities; specific groups who may be hard to reach; suggestions to promote MDA in the future in their communities.

### Translation of instruments

Translation of the questionnaire followed a uniform multi-stage process in all five countries to ensure consistency and allow for later aggregation of the data. First, two translators independently translated the survey from English into the local language. Next, the two translators compared their two versions of the survey, identified any discordant translations and resolved inconsistencies before agreeing to a final version. A third translator then translated the questionnaire from the local language into English (back translation). The three translators made further revisions together and agreed on a draft version for field-testing. During the training of the enumerators, the draft survey was tested in the field and final minor revisions were made according to that feedback. Topic guides for FGDs and IDIs were translated directly into the local language and reviewed for clarity and consistency. They were field tested and language was adapted as needed.

### Sampling

The selection of the communities or localities where the acceptability study would occur was based on the safety trial time schedule for that country. The window for selection into the acceptability study was within four months of drug ingestion during the safety trial to minimize recall bias. As a result, some localities were excluded from the acceptability study if they fell outside of this four-month period. Once localities were identified, sampling for the acceptability survey was performed by randomization of participants from the parent clinical trial using a sampling frame stratified by drug regimen and sex. The goal was to include 200 participants per study arm for a total of 400 individuals per country for Haiti, Indonesia, India and PNG. In Fiji, the goal was 150 participants per study arm (n = 300). Fiji had a different sample as there were three study arms in the Fijian study: two arms (n = 150 each) mirrored the other four countries (IDA and DA), while a third arm (n = 150) included a further treatment of permethrin following the IDA treatment. As such, this third arm was excluded from the 5-country aggregate analysis. Enrollment of participants followed a two-step process. First all individuals 14 years and older with a positive test for the presence of microfilariae (Mf) were purposefully included in the sample. All Mf positive individuals were included within the selected communities with the anticipation that the aggregate study would have 125 infected individuals in each study arm. Then a random selection of negative individuals from the selected communities made up the remainder of the cohort. We ensured that this selection mirrored the gender balance for that location as per the safety trial gender balance.

Only individuals aged 14 years and older were included in the study. Barcodes generated for each participant during the safety trial provided the link between the clinical data, evaluation of adverse events and the acceptability data.

Focus group discussions were performed with members of selected groups whose characteristics were expected to influence treatment acceptability. These included groups of young people, older men and women, community drug distributors and community leaders. All FGDs occurred in communities receiving IDA to explore specific themes related to the addition and use of ivermectin. In-depth interviews (IDI) with key informants were carried out with influential individuals in the study villages, including those who had participated in MDA during the safety trial or in past MDA. Individuals from both IDA and DA villages were included in the IDIs. These individuals were purposively identified based on their roles as opinion leaders, regardless of their participation in the safety trial. Participants for the FGDs and the IDIs were identified from the same villages where the acceptability surveys were administered.

### Training

Enumerators were recruited locally and did not participate as staff for the safety trial. Training consisted of a series of presentations, role plays and in-field observation. Enumerators were informed about the background of the study, the importance of obtaining informed consent, questionnaire administration and professional conduct while performing the survey. The training methods were consistent between sites with slight modifications to adapt to the local context.

### Data collection

Survey data collection was conducted using paper questionnaires in India, Indonesia and Papua New Guinea, and then entered into a database using Research Electronic Data Capture software (REDCap) [37,38] via a mobile application. In Haiti, data collection was performed by entering participants’ responses to the survey directly into the REDCap mobile application. Fiji combined paper questionnaires and online data capture. All FGDs and IDIs were performed at a location convenient to the participants’ homes using standardized topic guides. Audios of the FGDs and IDIs were recorded for later transcription and translation into English. All uniquely identifiable information was removed at the time of transcription.

### Analysis

#### Quantitative

The primary objective of this study was to assess the overall acceptability of IDA as compared to DA. To assess this outcome, a composite acceptability score was created by summing the values of nine acceptability indicators that were each scored on a four-point scale (Table 1).

**Table 1:** *Description of nine acceptability indicators*, *combined to make the acceptability score*.

Number	Indicator
1	These drugs work against LF
2	These drugs work against itching
3	These drugs work against intestinal worms
4	I would take this treatment again
5	I would recommend this treatment to my relatives
6	I would be willing to change my family’s routine so that we take the treatment again
7	I liked this treatment
8	This treatment is a good way to help our health problems here
9	Overall, this treatment will help my community

Notes: Participants answered along the continuum of disagree a lot, disagree, agree, agree a lot.

The range of acceptability scores was 9–36 and the median, 22.5, indicated the threshold of acceptability. Therefore, an individual score (or mean score for the group) above 22.5 was considered as the threshold of acceptability. Using the median followed the methodology of the Intervention Rating Profile tool to measure treatment acceptability in education whereby the median represents a level of moderate acceptability [12].

These data were analyzed using univariable and multivariable linear mixed models that included a random effect to account for possible clustering among participants within villages. In addition to the MDA intervention, our models included those variables that were collected within a week of treatment that are thought to influence acceptability including: sex, the presence of an AE following treatment, pre-treatment microfilaria (Mf) status, and participant age (adults vs children [14–17 years]). As a sensitivity analysis, we also explored whether treatment effects varied by country by assessing a treatment-by-country interaction. In addition to the primary research objective, we also compared key variables of interest between the different treatment groups (DA vs IDA), and explored inter-country differences for the constituents of the aggregate acceptability scores (i.e., subscores). Student’s *t*-tests, chi-square tests, or Fisher’s exact tests were used as appropriate. All analyses were conducted using SAS version 9.4 (SAS Institute Inc., Cary, NC, USA) and *P*-values < 0.05 were considered significant.

In the study design, we hypothesized that AEs would be associated with lower acceptability. To assess the potential relationship, we linked the acceptability survey participants through a shared barcode to clinical data on AEs collected by the safety trial teams [11]. This allowed for assessment of the perceptions of AEs versus the clinical assessments.

#### Qualitative

Focus group discussions (4–8 per country) and IDIs (8–10 per country) with key informants were conducted. Qualitative data were not linked to data from the clinical trial; however, survey participants were drawn from the same villages. Verbatim transcriptions of the digital recordings were done and then translated into English by the national research teams in each country.

Emergent themes and their respective sub-themes were captured from the verbatim transcripts, which were subsequently arranged in a taxonomy for analysis. Members of the research team reviewed the same transcripts to ensure inter-rater reliability. When discrepancies arose, the team discussed and came to consensus on the theme and its criteria for placement within the taxonomy.

Qualitative data were analyzed using NVivo version 11.4.1.

### Study locations

The study locations within each country corresponded to those in the parent IDA safety study [11]. Table 2 outlines the history of MDA in each location, the LF species and the settlement types.

**Table 2:** *Description of study locations*.

Country	District/Study site(s)	Total number of localities selected for study	Settlement type	Species of lymphatic filarial parasites	History of MDA rounds, using DEC + Albendazole prior to safety trial
Fiji	Rotuma Island and Gau Island	23	Remote; rural	*Wuchereria bancrofti*	>10 rounds
Haiti	Quartier Morin, Departement de Nord	10	Semi-urban	*Wuchereria bancrofti*	8
India	Yadgir District, Karnataka State	4	Semi-urban	*Wuchereria bancrofti*	12
Indonesia	Sumba Barat Daya District, Nusa Tenggara Timur Province	12	Remote; rural	*Wuchereria bancrofti*.*; Brugia timori*	No history of MDA
Papua New Guinea	Bogia District, Madang Province	7	Remote; rural	*Wuchereria bancrofti*	No history of MDA

### Ethics

At the time of the community safety study, all participants were informed about the upcoming acceptability study and in the safety trial consent, agreed to be contacted for the purposes of that study. Prior to recruitment into the acceptability study, written consent was obtained for all individuals selected to participate. Individuals unable to sign their name provided evidence of consent using their thumbprint and a signature from a witness confirming that the information sheet had been read. Information sheets with details of the study were provided for all participants. Participants <18 years of age required written parental consent. Focus group and in-depth interview participants were also asked to consent digital recording of the discussion.

## Results

### Surveys

A total of 1919 participants were included in our analyses. (S1 Table) The distribution of males and females, Mf and FTS positivity, AEs, and age was similar between the treatment groups. (Table 3)

**Table 3:** *Comparisons of demographic*, *infection and adverse event profiles of study participants across treatment groups*.

Variable	DA (N = 907)	IDA (N = 1012)	*P*-value
Female	47.5% (431/907)	47.7% (483/1012)	0.927
Adults (≥ 18 yrs)	86.3% (783/907)	85.6% (866/1012)	0.635
Mean Age (years)	35.2±16 [907]	33.6±15.2 [1012]	0.021
Mf Positive*	12.0% (109/905)	12.4% (125/1008)	0.812
FTS Positive*	27.9% (253/906)	27.3% (276/1011)	0.760
Any Adverse Event*	18.4% (161/874)	16.2% (162/997)	0.215

*Notes*: Reported as mean ± standard deviation [n] or n/N (%); all *P*-values generated from t-tests for continuous variables and chi-square or Fisher’s exact tests for categorical variables.

*Abbreviations*: Mf = Microfilaria; FTS = filarial test strip

* missing values

Filarial infections were more common in males than in females (Mf positive, 17.7% vs. 6.2%, *P* < 0.001; CFA positive, 36.2% vs. 18.2%, *P* < 0.001). (Table 4)

**Table 4:** *Demographic characteristics by gender*.

Variable	Group	Male	Female	*P*-value
Mf^	Mf (-)	82.3% (823/1000)	93.8% (856/913)	< .001
	Mf (+)	17.7% (177/1000)	6.2% (57/913)	
Mean Mf Count *		10.1 (8.1, 12) [177]	7.4 (5.2, 11) [57]	0.152
FTS^	Negative	63.8% (640/1003)	81.8% (748/914)	< .001
	Positive	36.2% (363/1003)	18.2% (166/914)	
Maximum AE Grade (among individuals with any AEs)	Grade 1	90.7% (137/151)	87.8% (151/172)	0.397
	Grade 2 or higher	9.3% (14/151)	12.2% (21/172)	
Mean age in years		35.3±15.8 [1005]	33.3±15.2 [914]	0.003

*Notes*: Reported as mean ± standard deviation [n] or n/N (%); all *P*-values generated from t-tests for continuous variables and chi-square or Fisher’s exact tests for categorical variables.

*Definition*: Grade 1 = mild, participants can go to school or work; Grade 2 = moderate and interfere with work or school; Grade 3 or higher = required evaluation by medical staff.

*Abbreviations*: Mf = Microfilaria; AE = adverse events; FTS = filarial test strip

* Log-transformed Mf counts for Mf positive samples were analyzed and are reported as geometric mean (95% CI) [n]

^ Missing values

Reported rates of past participation in MDA varied in countries that had previously provided MDA. In India, 85.1% (338/397) of survey participants reported that the safety trial was their first LF MDA, while corresponding rates for Haiti and Fiji were 24.8% (101/407) and 15.2% (46/302), respectively. For participants from Indonesia and Papua New Guinea, the safety trial represented their first exposure to mass LF treatment.

### In depth interviews and focus group discussions

Twenty-seven FGDs and 42 IDIs were conducted. A breakdown of participant characteristics and the number of sessions conducted in each country is provided in Table 5.

**Table 5:** *Breakdown of in-depth interview and focus group discussion participants for all five countries*.

	In-Depth Interviews (N = 42)		Focus Group Discussions (N = 27)	
Country	Number of Interviews	Participants	Number of Focus Groups	Participants
Fiji	8	Women’s leader, Nurse, Government leader, Religious leader, Nurse, Doctor, and Village chiefs	8	Community Health Workers (x2), Teens, Women’s Group, Village Headmen/Chiefs (x2), Elderly, and Men
Haiti	8	Community nurse, elected officials, Religious (church and non-church) leaders, Local doctor, Community drug distributor	4	Community Drug Distributors, Men, Women, and Young Men
India	10	Community drug distributors, Community leaders, Village chiefs	6	Health Staff (x2), Men’s group (x2), Women’s group (x2),
Indonesia	8	Health officials, Nurse, Elected officials, Village chief, and Religious leader	5	Community leaders, Community health workers (*kader)* (x2) and Women’s Group, Men’s group
Papua New Guinea	8	School administrators, Health officials, PNG IMR staff member, Elected officials, Local leaders, Women’s leader, President and two Female Community Health Workers	4	Women’s group, Community leaders (x2), Young men’s group

The analysis process yielded 13 emergent key themes related to the participants’ views and experiences: implementation of the intervention, awareness, knowledge, risks, lived experiences with treatment, community context, non-compliers, perception of burden of disease, anticipated benefits to personal health, trust, values, emotion, and adverse events.

### Quantitative analysis of acceptability scores

**Fig 1:**
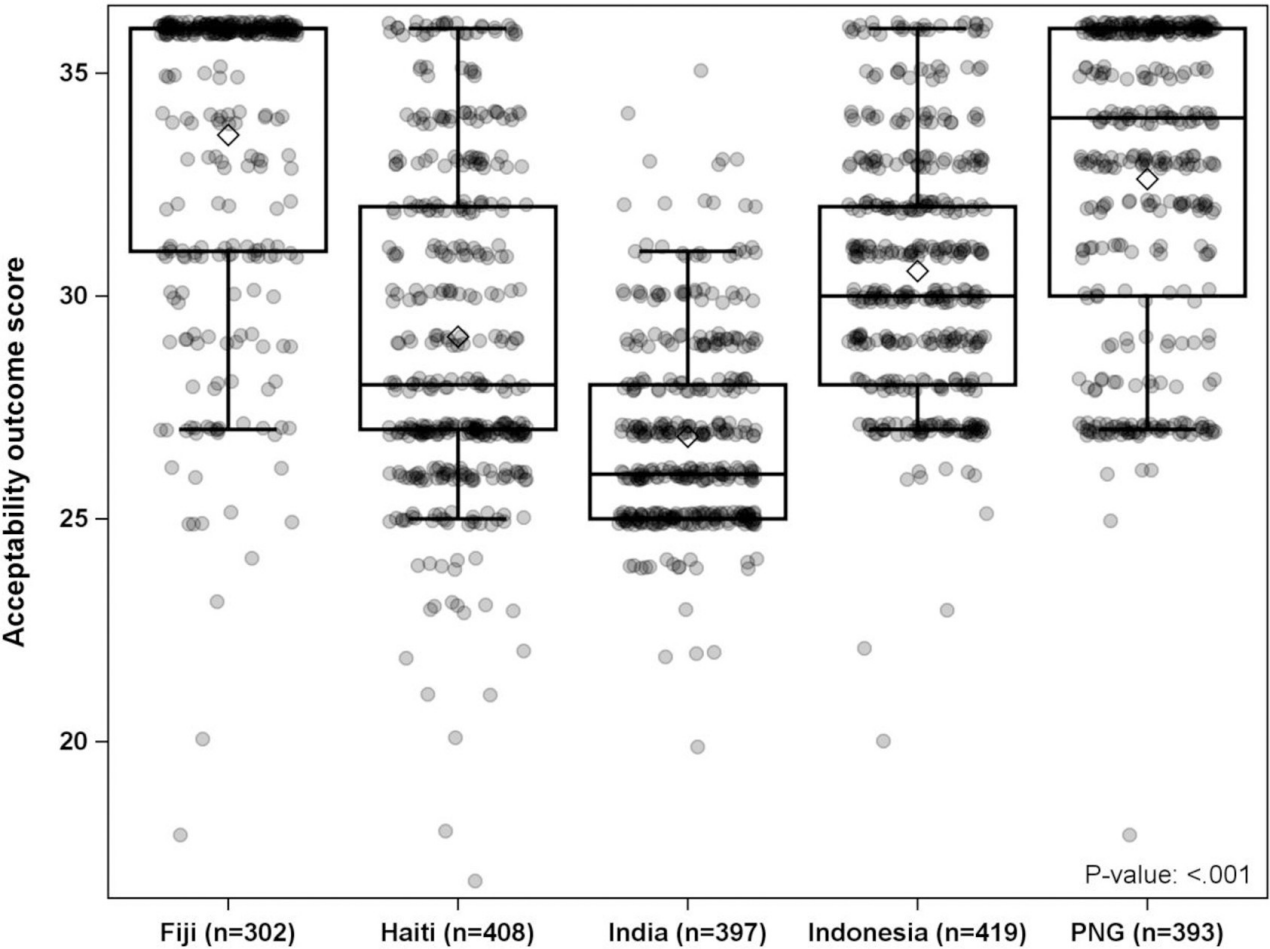
Aggregate acceptability scores for all five countries included in the study. Box plots include the mean (diamond), median (horizontal line), interquartile range (upper and lower edges of box), and whiskers (1.5 x IQR beyond upper or lower quartile). Scatter plots of the individual data points are overlaid with data randomly jittered to reduce overlap.

The acceptability scores for IDA and DA were similar in the unadjusted model (DA–IDA mean 95% CI: -0.1 [-1.6, 1.3], *P =* 0.83) and in the adjusted model (DA–IDA mean 95% CI: 0 [-0.7, 0.7], *P =* 0.95). Acceptability was not significantly associated with age, recorded clinical AEs or Mf positivity. Sex and country effects were both significant in the univariable and multivariable models. Females had lower acceptability (mean and 95% CI: 30.3 [29.8, 30.7]) compared to males (mean and 95% CI: 30.6 [30.2, 31.1]) (*p =* 0.007 for sex difference). All countries had MAS above the threshold of 22.5, indicating that MDA was acceptable across countries. (Table 6, Fig 1).

**Table 6:** *Results from univariable and multivariable linear mixed model analyses of the aggregate acceptability score outcome*.

Variable	Group	Univ Mean (95% CI)	Univ *P*-value	Univariable N	Multivariable Mean (95% CI)	Multivariable *P*-value	Multivariable N
Adult	Adult (> = 18 yrs)	31.4 (30.7, 32.2)	0.502	1919	30.4 (29.9, 30.8)	0.477	1865
	Child (< 18 yrs)	31.6 (30.7, 32.4)			30.5 (29.9, 31.1)		
Any adverse events	No	31.4 (30.7, 32.2)	0.988	1871	30.4 (30, 30.9)	0.951	1865
	Yes	31.4 (30.6, 32.2)			30.5 (29.9, 31)		
Country	Fiji	33.7 (33.1, 34.3)	<0.001	1919	33.6 (33, 34.3)	<0.001	1865
	Haiti	28.6 (27.8, 29.4)			28.5 (27.7, 29.4)		
	India	26.8 (25.6, 28)			26.8 (25.5, 28)		
	Indonesia	30.6 (29.8, 31.3)			30.4 (29.7, 31.2)		
	Papua New Guinea	32.9 (31.9, 33.8)			32.8 (31.9, 33.8)		
Drug regimen	DA	31.4 (30.3, 32.4)	0.835	1919	30.5 (29.9, 31)	0.946	1865
	IDA	31.5 (30.5, 32.5)			30.4 (29.8, 31)		
Sex	Female	31.3 (30.5, 32)	0.013	1919	30.3 (29.8, 30.7)	0.007	1865
	Male	31.6 (30.8, 32.3)			30.6 (30.2, 31.1)		
Mf test results	Mf (+)	31.3 (30.5, 32.1)	0.442	1913	30.3 (29.7, 30.9)	0.136	1865
	Mf (-)	31.5 (30.7, 32.3)			30.6 (30.2, 31.1)		

*Notes*: Univariable models include only a single fixed effect and a random effect for locality, and the multivariable model includes all fixed effects and a random effect for locality. Estimates correspond to model-adjusted means and 95% CI.

Acceptability of the treatments was highest among participants in Fiji with a mean acceptability score of 33.7 (95% CI: 33.1, 34.3), where over 58% of participants chose the highest possible acceptability score of 36. Similarly, the mean acceptability score was high among participants in PNG, with a mean of 32.9 (95% CI: 31.9, 33.8). Treatment acceptability was lowest in India with a mean acceptability score of 26.8 (95% CI: 25.6, 28). Mean acceptability scores among participants in Haiti and Indonesia were 28.6 (95% CI: 27.8, 29.4) and 30.6 (95% CI: 29.8, 31.3), respectively. Results of the multivariable analysis of acceptability were similar (Table 6) and the ICC for locality from the multivariable model indicated that locality accounted for about 13% of the variability in the data. Although there were large differences in acceptability scores between countries, we did not find evidence of differential treatment effects between countries (*P =* 0.54 for a Country x Treatment interaction). A table of coefficients from the multivariable linear mixed model are available as a supplement. (S2 Table)

When the scores for each of the nine indicators of the total acceptability score were analyzed, marked differences between countries were found. (S3 Table) The country with the highest rate of the ‘disagree’ response was India. For the variables that corresponded to itching and intestinal worms, greater than 84% of subjects in India disagreed that the MDA helped with these conditions, contributing to lower overall acceptability. This contrasts with the other countries where the overwhelming majority responded ‘agree’ or ‘agree a lot’ to these questions. For the acceptability question pertaining to whether a participant thought that the MDA was a good way to help their community’s health problems, India had the highest percentage that disagreed (35.3%) whereas over 98% of subjects in the other countries selected ‘agree’ and ‘agree a lot’.

### Combined results related to acceptability

The presentation of these data combined results from both the survey and qualitative interviews sub-grouped along four components of acceptability: product formulation, presence of AEs, how the intervention (MDA) was implemented and alignment with personal perceptions and values (including anticipated benefits to health and emotional response). Since there was no difference in acceptability detected between the IDA and DA arms, the results were presented as combined from both arms.

### Product formulation and application

Responses regarding the number of pills varied significantly across the five study sites. Study participants in Indonesia were happiest about the number of pills (87.1% [365/419] were very happy / happy) whereas participants in India were the least happy, with 52.9% (210/397) feeling unhappy or very unhappy. 55.0% (166/302) of participants in Fiji had the highest number of “neutral” feelings. (Table 7)

**Table 7:** *Feelings about number of pills*.

Variable	Fiji (N = 302)	Haiti (N = 408)	India (N = 397)	Indonesia (N = 419)	Papua New Guinea (N = 392)*	Total (N = 1918)		
	Freq (%)	Freq (%)	Freq (%)	Freq (%)	Freq (%)	Freq (%)	*x*^*2*^	P-value
Very happy	64 (21.2%)	142(34.8%)	30 (7.6%)	272 (64.9%)	121 (30.9%)	629 (32.8%)	971.1	<0.001
Happy	59 (19.5%)	169 (41.4%)	40 (10.1%)	93 (22.2%)	159 (40.6%)	520 (27.1%)		
Neutral	166 (55.0%)	76 (18.6%)	117 (29.5%)	46 (11.0%)	71 (18.1%)	476 (24.8%)		
Unhappy	7 (2.3%)	16 (3.9%)	184 (46.3%)	7 (1.7%)	34 (8.7%)	248 (12.9%)		
Very unhappy	6 (2.0%)	5 (1.2%)	26 (6.5%)	1 (0.2%)	7 (1.8%)	45 (2.3%)		

* 1 missing from PNG

The qualitative study provided similarly variable results regarding the number of pills across the countries. Some participants were not happy about the number of pills; however, when they understood the value of the treatment, they were willing to take the treatment regardless of the number of pills. This also extended to individuals in the DA arm requesting the addition of ivermectin.

“*The medicine itself is good so one thing that I personally feel that it’s not good is the number of medicine that we are taking*, *it is a lot*.*”—Village Leader FGD*, *Papua New Guinea*

“*Some we held onto the medicine and we said*, *“Eh*, *twelve tablets is too much*,*” but we just drink as we [are] concerned about our life*.*”–Women’s FGD*, *Papua New Guinea*

“*Some people complain about it*, *some people said it was too much*. *I’m usually scared of pills but I took them*.*”–IDI with Nurse*, *Haiti*

“*You know that Haitians do not like to take pills*. *If they don’t know anything about the disease they won’t take it*, *but if they know the consequences*, *they will*.*”—Young Men’s FGD*, *Haiti*

“*They had to take the medicines in front health personnel*, *not otherwise*. *Since that was the case*, *no matter the number*, *they did take them*.*”–Community Health worker FGD*, *Indonesia*

“*And when we in [my village]*, *we were about to take the third tablet*, *they told me that they want to take it too*. *And I told them they were the ones to take the 2-drug medication*. *It was decided before in the programme but I will put their request when we next have a meeting*. *They want to take the third tablet*.*”—Community Health Worker FGD*, *Fiji*

### Implementation of the intervention (mass drug administration delivered via safety trial)

The safety study was different from routine MDA, as treatment was provided by doctors and/or nurses, testing was done prior to treatment and active and passive AE monitoring was done. For those communities who had experienced community-directed MDAs before, these differences were reflected in the qualitative data. In the analysis, comments related to implementation were grouped in themes reflecting the professionalism of the drug delivery team, the manner with which the drugs were distributed (directly observed therapy) and overall trust in the team.

For many study participants, their comments demonstrated appreciation for the professionalism of the MDA, especially regarding the conduct, appearance and patience of the distribution team. Although all sites highlighted elements of professionalism in the teams, these comments were more frequently identified in those sites with prior MDA experience (Haiti, Fiji and India).

“*Anganawadi people (community drug distributors) will not look or care for us*. *But you people showed care*, *so we like your tablets*. *You people formally asked us first*, *then gave us the tablets*.*”—Women’s FGD*, *India*

“*You people are giving so much attention and working with greater responsibility*. *We are really thankful to you*.*”—Men’s FGD*, *India*

“*I can say it is acceptable because the place where the pills were given was clean*, *that means it is well done*, *they don’t even touch the pills*.*”—Men’s FGD*, *Haiti*

“*We are happy about it*. . . . *the awareness team came and explained… about the drugs*, *a lot of other things to us*… *The team came and did the work*, *we consumed the drugs*, *not just that…we are happy with the care provided to us*.*”—Women’s FGD*, *Fiji*

The involvement of doctors was appreciated by those who participated in the trial in India. This represented a change from previous MDAs in which the regular distribution carried out by local community workers in the villages called *Aganwadi* and *Asha*.

“*Why should we take these pills*? *Something wrong may happen*. *We do not have the elephantiasis and so we will not take these pills*, *they would say*. *When the doctor told them in detail*, *they agreed to take it and they took it*.*”—IDI female community health worker*, *India*

“*This year we have confidence in doctors*, *so we took it*.*”—Women’s FGD*, *India*

“*You people are not like Anganwadi workers*. *Doctors gave us phone numbers to contact after taking the tablets*. *We have trust in your team…Sorry*, *we don’t trust [Anganwadi workers] in this matter*. *If they come with doctors we will take*, *otherwise we will not take tablets*.*”—Women’s FGD*, *India*

Participants of the FGD and IDI noted the shift to directly observed therapy that was part of the safety trial. Participants from countries where MDA had been administered before preferred this change to the previous implementation of the distribution, and interpreted it as increased attention, responsibility and care from the delivery team.

“*This new way is better because the tablets are given and the person takes them right in front of everyone and we see it happen but with the first (distributions) the person takes the tablets home and we’ll never know*. *I like this one and everyone likes it too because all who came took the medication*.*”—Village Chiefs FGD*, *Fiji*

“*We don’t let them go*. *We talk to them to let them know that the pills are not a problem and that they can drink it but sometimes some people say that they can’t*. *But after eating and before sleeping they take them*. *It’s not a problem*.*”- IDI with Nurse*, *Haiti*

“*In previous program*, *responsibility of Anganwadi workers was only up to providing tablets*. *But now they give tablets and they ask to take tablets in front of them*. *So these people take full responsibility*.*"—Men’s FGD*, *India*

The qualitative studies identified trust as a theme that overlapped with professionalism. Trust appeared to be a critical factor for treatment acceptance, especially in Haiti and India.

“*If they didn’t trust them they wouldn’t come in mass*.*”—Women’s FGD*, *Haiti*

“*Maybe the next time*, *(the ones who refused) will take it*. *I think maybe they do not have that trust in the programme like those who took the medication*. *Maybe they have seen what happened and they will take it in the next round*.*”—Community Health Worker FGD*, *Fiji*

“*The distribution happened in the yard of my house*. *It happened in the area but I wasn’t present*, *I went to play soccer*. *When I came back*, *I saw a lot of people*, *I didn’t know what was happening then I saw they were giving pills*, *I didn’t have the intention to take them but when I saw how they were taking care of the population*, *it was really different from the past years*, *I decided to take them*. *I also called friends to do so because they didn’t know*.*”—Haiti young men’s FGD*

IDIs and FGDs also revealed several factors that might influence an individual’s trust in a LF control program. These factors included, but were not limited to, the conduct and identity of the distributors, the information provided to community members, involvement of community leaders and government officials, community members’ awareness and knowledge of the distribution, and rumors that had been spread about the distribution.

“*Yes we have taken these pills*. *But this is the first time that we have taken these pills*. *Because we have faith in you and that is why we have taken it*. *Because you all came and you conducted blood tests and checked whether we are positive or negative and then when you told us to take the tablets*, *you gained our confidence and because of that we have taken these pills*.*”—IDI community leader*, *India*

"*One thing I liked about the distribution is that the people involved were very patient"—Women’s FGD*, *Haiti*

"*I liked that they visited and provided information"—Women’s FGD*, *India*

"*People understand the importance of this recent LF drug distribution especially when the results are explained to them*.*"—Community Health Worker FGD*, *Fiji*

"*We are happy about it*. *As I had mentioned*, *staying like this in the village*, *the awareness team came and explained*, *explained about the drugs*, *explained a lot of things to us women*.*"—Women’s FGD*, *Fiji*

Several contextual factors were also discussed as potential influencers of a community’s trust in the distribution that went beyond previous MDA programs. For example, in Sumba Barat Daya District in Indonesia some individuals had previous negative experiences with people selling ineffective medications for other diseases. These experiences led to some doubts about the legitimacy of distributed treatments of any kind.

“*Here*, *as far as I work*, *most of them are like that*, *because there are people that come here selling medicines*, *like mobile doctors; they sell medicines and the community pays so much*. *But they remain ill*, *never healed*. *That’s where the perception comes from*: *we take the medicine means we will remain ill and we will not have children any longer” IDI with Nurse*, *Indonesia*

Some participants reflected on their previous experiences with community MDA and the reasons why they did not participate in the past.

“*I never trusted them*. *They used to give them at school*. *Either I was at school or not*, *I didn’t trust them*. *I always put the pills somewhere*, *they can stay here for years*.*”*

“*Why you never trusted them*?*”*

“*Because I didn’t like the way they were distributing the pills*. *There are pills for different age and they don’t really know your age*.*”—Young Men’s FGD*, *Haiti*

Overall, most survey participants (94.1% [1805/1919]) responded that they had a lot of trust or confidence in the drug distributor used in the safety trial. There was little difference in trust between countries (range 91.2% - 97.4%) or sex of the participants (females 94.3% [862/914] and males 93.8% [943/1005]). Participants who reported taking the LF treatment for the first time during the safety trial had more trust in the distributors (95.2% [1220/1282]) than participants who had previously participated in the MDA (91.8% [584/636]) (P = 0.006).

### Post-treatment adverse events

Interventions that have AE are likely to be less acceptable than those that do not (25). As noted above however, the clinical presence of AEs was not associated with the mean acceptability score.

Overall, 17.3% (323/1871) of the acceptability survey participants had clinically assessed AEs during the post-treatment monitoring period as evaluated by the safety study teams (48 missing people related to missed AE assessment in the safety study). Female participants had higher presence of clinical AE (19.2% [172/897]) compared to men (15.5% [151/974]) (P = 0.04). When surveyed four months later during the acceptability study, 43.3% (140/323) of participants who had clinical AEs recorded during the safety study reported that they did not recall having experienced any AEs following treatment. Furthermore 26.7% (413/1548) of those with no clinically assessed AEs during the safety study reported that they had experienced an AE following treatment.

Most (68.3% [1311/1919]) study participants reported that they had not experienced any AEs in the day following treatment, with some variation across the sites: India (88.7% [352/397]), PNG (77.1% [303/393]), Fiji (69.2% [209/302]), Haiti (55.2% [225/408]) and Indonesia (53.0% [222/419]) (P<0.0001). The perception of a low number of AEs was echoed in the qualitative transcripts:

“*All the people took tablets*, *no serious problems*. *We are all fine*.*”—Men’s FGD*, *India*

“*Oh yeah*! *I’ve got three pills; there are three pills I got*, *I did not feel any effect of the drug after that*.*”–IDI with key informant*, *Papua New Guinea*

“*We didn’t feel anything*. *It was like a treatment from a prescription of a doctor*. *I didn’t feel anything*.*”–Men’s FGD*, *Haiti*

“*Doctors have given 5–6 pills after checking their weight and height*, *still people were talking that the number of pills were more*. *People thought something may happen if so many tablets are taken at once*, *but no one suffered anything*.*”–IDI with community leader*, *India*

“*When we took the medicine*, *we did not feel anything*. *We went to bed and felt good*.*”–IDI with community health worker*, *Indonesia*

Despite most participants reporting that they did not have any AEs post treatment, 21.3% (409/1919) of the people included in the survey reported that someone in their family had AEs post treatment and 60.0% (1142/1903; 16 missing) reported that there were people in their community who had experienced AEs.

Participants often reported improvements in their own physical state following treatment. Survey participants in Fiji and Indonesia reported the highest improvements in skin sores (12.3% [37/302] and 12.9% [54/419], respectively), itching on the skin (13.3% [40/302] and 17.9% [75/419], respectively) and itching on the head (9.9% [30/302] and 13.6% [57/419], respectively). Participants across all countries reported improvements in appetite (30.5% [585/1919]), quality of sleep (28.3% [543/1918; 1 missing]), physical strength (23.0% [442/1919]) and energy levels (22.1% [423/1918; 1 missing]) (more than one response was possible).

“*I really wanted to sleep*. *There are many of us in our district feeling that we wanted to sleep and I had a really good sleep*. *The next day I felt really fit*.*”—Community Health Worker FGD*, *Fiji*

“*Yes*, *side effects*. *It was nice for these people to be sleepy*. *They were happy about being sleepy*!*”–Village Headmen FGD*, *Fiji*

“*I myself experienced itching on my hands (showing hands)*. *…Well the itching got worse after taking the treatment and I feel it has since improved slowly since then*.*”–Village Headmen FGD*, *Fiji*

“*Community here*, *as I have explained*, *prior to being informed*, *they were afraid and nervous*. *But they have taken the medicine and they have said they felt quite good*. *And they were very pleased because the germs*, *worms*, *came out*.*”–IDI with community leader*, *Indonesia*

"*Man came and said that he has this itchiness that makes him scratch all the time and after he had taken the tablets he felt relieved*. *Like his skin became smoother*. *The itchiness is sort of no longer there*.*"—FGD*, *community leaders*, *Fiji*

"*The itch has gone*. *Before taking the medicine*, *I felt itchy*. *But after taking the medicine*, *it’s gone*.*"—IDI with community health worker*, *Indonesia*

Participants report symptoms that were generally consistent with the AEs expected from the treatment: cold/chills, irritability, drowsiness, dizziness, fatigue, headache, fever, weakness, body/stomach pain, decreased appetite, and diarrhea, swelling and itching (11). Surveyed females reported AEs more than males (35.6% [325/914] vs 28.2% [283/1005]; P = 0.01), including more nausea (P = 0.002) and headache (P<0.001). Male participants felt better more often after treatment than female participants did (10.1% [101/1005] vs 5.0% [46/914]; P<0.001). This was consistent with the difference in clinically assessed AE between males and females presented earlier.

There was no difference by sex in those who reported passing worms after treatment. Indonesian participants reported a higher rate of passing intestinal worms following treatment (22.4% [94/419]) as compared to the other sites (combined 0.86% [13/1499; 1 missing]) (P<0.001).

“*Some people have experienced some vomiting and headache and minor side effects only*. *No other problems*.*”–IDI with community leader*, *India*

“*I’ll be honest*. *When I took the medicine*, *first I felt drowsy and dizzy but after that I felt easily angered*.*”–IDI with Nurse*, *Indonesia*

“*I was sick and I vomit after taking the medicines*. *After I took the medicines I came*, *sit and watch and then I vomit*. *It was a foul vomit*, *one of my buddies said the medicine has cleansed my body*. *I thought that the medicine was going to kill me*.*”—Young men’s FGD*, *Papua New Guinea*

“*The bad thing about this treatment is the side effects that affects people*. *It slows down the energy of some men in the community*, *it weakens their body and they feel that they don’t have the same level of energy to do the work that they used to do*, *the medicine they took has reduced their energy*. *They felt sluggish*, *they felt like sleeping*, *some they went and work for a while*, *they felt hungry and came back*.*”–Young men’s FGD*, *Papua New Guinea*

The potential for AEs concerned some community members (28.5% [546/1919] responded yes). 39.7% participants (762/1919) reported that they were not personally concerned about AEs. Some individuals understood that AEs were caused by the medicines working to kill the parasites. Several participants reported accepting the treatment despite being concerned about (or having experienced) AEs.

“*Some of us were a bit scared but the staffs [sic] told us you will be feeling this and that*. *When you are having such feeling you must know that the medicine is working inside your body to kill these germs*. *They were telling us this and encourage us to take the medicine…They told us that you will feel dizzy*, *you will be feeling drowsy and so when we experienced it we knew straight away*, *that was what they were telling us*.”*–Village Leader FGD*, *Papua New Guinea*

“*After taking the medicine*, *there were some who were dizzy*, *some claimed to have general uneasiness*. *But after we explained to them or I explained the effect of the medicine*, *they feel satisfied*.*”–IDI with Nurse*, *Indonesia*

“*The person told me of being ill after taking the medicine*. *The person also told me that he did not have something to eat before but he was pleased to have taken the medicine to prevent the disease*.*”–IDI with Nurse*, *Indonesia*

In addition to the messaging, the approach to AE management was appreciated, particularly in those study sites that had previously received MDA.

“*There is nothing bad to say*. *It gave one of my kid fevers*, *diarrhea that’s why the doctor came and brought treatments*. *He assisted him before leaving*.*”–Women’s FGD*, *Haiti*

“*In case we experienced any side effect we received timely care from them by calling them*. *So there are no serious adverse events*.*”–Men’s FGD*, *India*

“*The rate of using these tablets was only 20 per 100 before*. *Now it is completely improved because of doctor’s presence at the time of taking the tablets*, *as there is nothing to fear about side effects*.*”–Health Staff FGD*, *India*

### Alignment with personal perceptions and values

For the treatment to be acceptable, it must also align with the personal perceptions and values of the individuals taking the pills. Related themes include perceptions of risk for LF infection, perceptions about the safety of the treatment, perceptions of the norms within the community as related to LF treatment and alignment of participation with an individual’s personal values.

The concept of “risk of infection” was captured through questions related to “personal concern for LF”, knowledge of others in the community who may have LF and those who are most likely to become infected. There was much variation across the five sites to the question of personal concern for LF. India (56.7% [225/397]) and Haiti (36.5% [149/408]) reported the highest numbers of survey participants who were not personally concerned about LF. Knowledge variables (transmission, cause, personal understanding of LF) may influence an individual’s perceptions about their own risk of infection. Across the research sites, there was variability in knowledge and understanding. (Table 8)

**Table 8:** Variables assessing survey participants’ understanding of the transmission, cause, and asymptomatic nature of LF across all five countries.

		Fiji (N = 302)		Haiti (N = 408)		India (N = 397)		Indonesia (N = 419)		Papua New Guinea (N = 393)		Total (N = 1919)	
		Freq	%	Freq	%	Freq	%	Freq	%	Freq	%	Freq	%
Self-rated understanding of LF*													
	No knowledge (1)	19	6.3%	73	17.9%	12	3.0%	122	29.1%	81	20.6%	307	16.0%
	A little (2)	10	3.3%	30	7.4%	23	5.8%	19	4.5%	25	6.4%	107	5.6%
	Average (3)	108	35.8%	234	57.4%	202	50.9%	131	31.3%	180	45.8%	855	44.6%
	Good (4)	11	3.6%	5	1.2%	17	4.3%	40	9.5%	46	11.7%	119	6.2%
	Very good (5)	149	49.3%	22	5.4%	35	8.8%	98	23.4%	54	13.7%	358	18.7%
	Don’t Know	0	0.0%	44	10.8%	108	27.2%	9	2.1%	7	1.8%	168	8.8%
**Mechanism of LF transmission**													
	Worms	1	0.3%	13	3.2%	14	3.5%	17	4.1%	38	9.7%	83	4.3%
	Mosquitoes	292	96.7%	285	69.9%	276	69.5%	82	19.6%	276	70.2%	1211	63.1%
	Water	3	1.0%	22	5.4%	48	12.1%	29	6.9%	5	1.3%	107	5.6%
	Curse	0	0.0%	0	0.0%	0	0.0%	2	0.5%	3	0.8%	5	0.3%
	Hereditary	0	0.0%	0	0.0%	5	1.3%	10	2.4%	24	6.1%	39	2.0%
	Other	1	0.3%	23	5.6%	0	0.0%	26	6.2%	5	1.3%	55	2.9%
	Don’t know	7	2.3%	95	23.3%	118	29.7%	295	70.4%	60	15.3%	575	30.0%
**Cause of LF**													
	Worms	2	0.7%	15	3.7%	16	4.0%	26	6.2%	169	43.0%	228	11.9%
	Mosquitoes	295	97.7%	279	68.4%	273	68.8%	64	15.3%	149	37.9%	1060	55.2%
	Water	5	1.7%	155	38.0%	126	31.7%	46	11.0%	10	2.5%	342	17.8%
	Curse	0	0.0%	0	0.0%	0	0.0%	2	0.5%	5	1.3%	7	0.4%
	Hereditary	1	0.3%	0	0.0%	6	1.5%	11	2.6%	28	7.1%	46	2.4%
	Other	1	0.3%	24	5.9%	0	0.0%	35	8.4%	4	1.0%	64	3.3%
	Don’t Know	4	1.3%	51	12.5%	117	29.5%	286	68.3%	52	13.2%	510	26.6%
**Can LF be asymptomatic^**													
	Yes	173	57.3%	212	52.0%	44	11.1%	62	14.8%	104	26.5%	595	31.0%
	Maybe	57	18.9%	26	6.4%	25	6.3%	36	8.6%	26	6.6%	170	8.9%
	No	60	19.9%	112	27.5%	229	57.7%	256	61.1%	201	51.1%	858	44.7%
	Don’t know	12	4.0%	58	14.2%	99	24.9%	65	15.5%	57	14.5%	291	15.2%

* 5 missing from Fiji

^5 missing from PNG

The FGDs provided additional depth to the understanding of individual concerns about LF. Participants described a range of individuals who are most at risk for infection with LF. Opinions varied across the five sites.

“*I think it’s the females*, *females get it more than males*. *That’s what I thought because from my perspective*, *counting the figures of the females it is more than the males*…*”—IDI with Community Health Worker*, *Papua New Guinea*

“*Men*, *mostly men*. *Not the children*, *mainly men*.*”–IDI with health provider*, *Papua New Guinea*

“*Most of the people that I know have filariasis are girls*. *They have big feet the most*.*”—Young Men’s FGD*, *Haiti*

“*I think that children are more unprotected*.*”—Young Men’s FGD*, *Haiti*

“*For elephantiasis*, *there is no gender bias*, *not even age is the factor*, *comes because of mosquitoes*.*”—Men’s FGD*, *India*

Some participants highlighted specific activities or environmental factors that they believed increased one’s risk of becoming infected with LF.

“*Normally men work outside i*.*e*. *in temples*, *fields*, *etc*. *so chances of mosquito bite are more in men*. *It is the reason that disease is more common in men*.*”—Health Staff FGD*, *India*

“*Here mostly people do not use footwear*, *wear no sandals*, *they just walk bare foot to the garden and raise their cattle and without being aware of it*, *they get the disease*.*”–IDI with community leader*, *Indonesia*

“*To have normal health*, *we should have a proper environment*. *The streets are not clean*, *the canals are dirty and full of mosquitoes*.*”—Young Men’s FGD*, *Haiti*

Finally, interview participants shared that one’s sense of personal risk for LF infection could influence their participation in the distribution.

“*We are thankful that the pills were given out to prevent the disease LF*. *Some of us may have the worms and we are happy about the distribution*.*”—Women’s Group FGD*, *Fiji*

“*I was never interested in taking the pills*. *I thought it was an old person disease because I usually see them with big foots*. *After consulting me they told me to come back at 9pm so they can give the pills because it was in my blood*.*”—Young Men’s FGD*, *Haiti*

In terms of burden of LF in the community, survey participants were asked to describe how many people in their own village had LF. The perception that there were many with LF living in the village ranged from the highest in India (30.2% [120/397]) to the lowest in Haiti (3.9% [16/408]). In the qualitative data, participants in India particularly remarked about the presence of LF in their village, commonly identifying the disease as one of the most prevalent in their communities. Whereas in Haiti, over half of survey participants (54.7%) reported that they didn’t know about the LF prevalence in their community, compared to 4% of participants who didn’t know in India.

### Anticipated benefits to personal health

Another important theme emerging from the qualitative data related to the benefits participants perceived they would receive if they participated in taking the LF treatment. Community members’ acceptance of treatment seemed to be related to whether they perceived the treatment as beneficial to their personal health.

“*So because of my status and hearing the awareness session*, *I was happy to take the pills in efforts to improve my itchy skin conditions*. *I have spent a lot of money going to Suva and Tamavua and in chemists for my medication*.*”—Village Headmen FGD*, *Fiji*

“*All people have taken because they felt that their health should be good*. *We have understood that if we don’t want elephantiasis then we should take these tablets*.*”—IDI with influential community member*, *India*

“*Those of us who went*, *we thought that it will protect us in the long run so we went ahead and drank the medicines*.*”—Young Men’s FGD*, *Papua New Guinea*

Most survey participants agreed (78.3% [1502/1919]) that the LF drugs were very important for their health. Across the five sites, India had the lowest percentage (58.4% [232/397]) and Fiji, the highest (86.4% [261/302]) in agreement with this statement. Men held this perception more than women in the survey (81.3% [817/1005] versus 74.9% [685/914]; P = 0.01). Survey participants were also asked how safe they felt the LF pills were. Most participants (72.4% [1388/1918; 1 missing]) felt that the treatment was very safe with a range from India (53.7% [213/397]) to Fiji (84.7% [255/301; 1 missing]). In India, 29.5% (117/397) reported that they didn’t know about the safety of the treatment. More men perceived the treatment to be very safe as compared to women (76.0% [764/1005] versus 68.3% [624/913; 1 missing]; P = 0.004).

### Emotional response to LF treatment

Discussions with health staff, government officials, community health workers, distributors and community members provided insight into what people valued about the distribution and what caused an emotional response. The aspects of the distribution that were most valued appeared to differ based on the participants’ role within their communities and their individual experiences. The theme of “emotion” was linked to any reference the interview participants made to their feelings regarding the distribution and/or LF. Happiness and fear were the most commonly expressed emotions amongst interviewees. Several people mentioned that their community was happy to have received medication that was provided during the safety study.

“*They are happy that the drug was made available at their door step*, *unlike mala-1*, *they don’t go looking for it*.*”–IDI with health staff*, *Papua New Guinea*

“*Most of the time it’s because the area is far from the city so when someone get sick*, *that person has to travel to find the right care needed and it’s a lot of spending*. *The person has the obligation to suffer because he can’t afford it but when health agent comes to them to give them the care they need*, *they are really happy and it is easier for them*. *If they are happy about the service*, *they always come back*.*”—IDI with community leader*, *Haiti*

“*Yes*, *for community*, *the service provided by the health team for treatment*, *the community is thankful because the team is from Jakarta and because it was free of charge*. *For the coming of the team we can only thank*, *because the service they provided is for health*.*”—Men’s FGD*, *Indonesia*

“*…what I am about to say a lot of people may not agree*, *but I didn’t see the distribution as something to make money in*, *it was because I wanted to*.*”—Community Drug Distributor FGD*, *Haiti*

“*I can’t really say anything but the things I was interested in*, *I felt proud that I felt proud to go out to motivate others*, *talk to them*, *laugh with them before giving them the treatment*.*”—Community Drug Distributor FGD*, *Haiti*

“*The community was very happy*, *they are happy because of this collaborative work*. *They did not just gave us and took off*, *they gave us and observed and check on us and they are still with us and this is much better*, *it’s effective according to our perspective*, *so I want it to be like that*.*”–IDI with community leader*, *Papua New Guinea*

“*We are really happy about the government as their health department gave so much attention and care*. *The team was so formal in their attitude as they asked children about their meal*, *provided us biscuit and chocolates with this care for disease*, *because of all these we are happy about this program*.*”—Health Staff FGD*, *India*

“*If there is treatment*… *we are happy*… *because we were told it is to prevent filaria*.*”—Community Leaders FGD*, *Indonesia*

Fear of AEs deterred some people from participating in MDA, however; fear was also seen as a powerful motivator. Several participants mentioned that the fear of becoming infected with LF compelled some people to comply with treatment.

“*Sometimes people are afraid to take the pills because they think that the pills give the same effects as the disease*. *Some sometimes notice swellings in their genital parts*. *Some people gain weight and people after hearing those are afraid to take the pills*, *if not more people would take it*.*”—Men’s FGD*, *Haiti*

“*Pictures of filaria*, *swollen foot*, *swollen breast and genitals*. *The pictures would encourage them because then they will be afraid*. *Once we explained to them with pictures*, *they said “Ah*… *it is better to take the medicine*.*” They were afraid*.*”–IDI with nurse*, *Indonesia*

“*Because of this sickness*, *being frightened of getting that sickness*, *they were very eager to receive the drugs to put a stop to it*.*”—IDI with community leader*, *Papua New Guinea*

## Discussion

The primary objective of this study was to understand whether there were any differences in acceptability of the triple drug regimen (IDA) compared to the standard two drug regimen (DA) amongst safety study participants in five countries. Our results showed that there was no difference in mean acceptability scores between the two drug regimens: DA or IDA. Acceptability was not associated with Mf presence, clinical AEs after drug consumption, education, age or source of income. Sex was shown to be associated with the mean acceptability score, with females having slightly lower scores than males. Acceptability was high across all sites, with marked inter-country variability. High acceptability scores were supported by the qualitative findings which highlighted some of the reasons why communities accepted the treatment regimens. Anticipated concerns about the higher number of pills and impact of potential adverse events with IDA were unfounded; largely because of the professionalism of the safety trial MDA and the attention to AE during the treatment period. These results are important for program implementation where IDA will be adopted as they reinforce the elements that communities appreciate in a quality MDA. Study results also support the use of IDA as an equally acceptable option for eligible LF elimination programs.

While the acceptability of the treatment was high overall, it was lower among female than male participants. It may have been related to differences in AE experiences and in knowledge. Female participants reported more AE than men did, both in terms of their perceptions of the AE they experienced as well as the clinical AE, as assessed by the safety study medical teams. These findings are consistent with the safety study data which also reported higher AE in females than in men [11]. In addition to differences in AE, it is possible that social mobilization to safety study communities may not have reached females in the same way it reached males. This may be explained by differences in participation of males and females in community meetings, one of the primary ways employed to inform communities about the safety study. As seen in other research, females may not attend these kinds of community meetings and if they do attend, may not actively participate in the meeting or the decision-making process [39].

Understanding the variation in acceptability across the five research sites can be explained by context, prior experience with MDA and confidence in how the LF treatment was delivered during the safety study. Each of the five study sites had variability in the provision and availability of health services. The research sites in PNG (Bogia District), Fiji (Rotuma Island) and Indonesia (Sumba Barat Daya District) were the most remote regions with the least developed health infrastructure when compared to the other two countries. As such, the provision of testing, diagnosis, free treatment and if unwell, a medical assessment by the safety study teams in the community, could have positively influenced the higher acceptability of both drug regimens in these communities. Even in India and Haiti, sites in which participants had better access to health care, the presence of medical staff in the villages while the LF treatment was delivered was appreciated.

The history of MDA varied across the five sites, ranging from MDA-naïve regions (PNG, Indonesia) to three sites with multiple years of MDA rounds (India, Haiti and Fiji). These experiences (or lack thereof) likely had positive effects on acceptability across the five sites. For those living in the naïve regions, the safety study and the treatment for LF were novel and addressed a disease that was evident in the community. For those living in Fiji, India and Haiti, it was the more professional approach to the distribution of LF treatment used in the safety trial that was particularly novel and noted by participants. This was reflected in the comments captured in the IDIs and FGDs. Comments about patience of the distributors, AE monitoring and cleanliness of the drug distribution team were particularly noteworthy. These elements to drug delivery may have succeeded in attracting individuals to take the LF treatment for the first time, despite being exposed to multiple MDA rounds in the past.

Part of this new approach to LF treatment included how information was shared with community members prior to the start of the distribution. In the three study sites with previous history of MDA, it is likely that social mobilization activities had diminished over time as the communities, community drug distributors and health providers became accustomed to the MDA and its messages. This may have had a particular impact on adolescents as they would have been school aged children when the LF elimination programs and associated social mobilization were launched. In contrast to routine MDAs, the purposeful approach to providing information about the treatment and its benefits during the safety study was appreciated across all age groups. Each site adapted their approach to providing information according to the local context. As such, the social mobilization carried out before the administration of either drug regimen was different in each of the five sites. The approaches to provision of information to communities ranged from radio announcements (Haiti), branded posters (Fiji), community outreach by community health workers (all sites) and community meetings with designated leadership (all sites). The social fabric, including the individualistic versus collectivist nature, of these locations may also influence how information is disseminated throughout the community [40]. The different approaches and contexts may have also accounted for some of the differences in acceptability seen across the five countries.

Across all sites, there was an appreciation for the professionalism of the delivery of the medications. This was particularly remarked by individuals living in those sites where MDA had occurred before. The use of directly observed therapy, the time the drug deliverers spent with the community members and the information provided were remarkable for study participants. The professional delivery of the MDA alleviated concerns about the larger number of pills delivered with IDA. Community members reported taking the large numbers of pills because they understood the value of the complete treatment.

Participants appreciated the strategies that were implemented to reassure and follow-up potential AEs. AE management depended on the context of the safety study, ranging from providing phone numbers for physicians to on-call health staff. All AE treatment was provided free of charge. This purposive approach to monitoring AE may have reduced the fear of AEs that trial participants could have had because they knew what to expect, where to go for help and had the assurance that help would be provided at no cost. In many contexts, the fear of AEs remains a frequently cited reason for refusal to participate in MDA [32,33,41]; however, this study showed that concern about AEs was relatively low amongst the study participants. As they were reassured about AE, it is possible that the memory of any experienced AE diminished over time and was even forgotten.

This is the first study we know of that compares recall of AEs with a clinical assessment of AEs following LF treatment. We observed discrepancies in the recall of clinical AE and perceptions of AE. There were also variations of individual perceptions, household perceptions of AE and community perceptions of AE. Individuals perceived that there were more AE in their communities than in their own households. This may reflect the community conversations about adverse events that may have augmented their importance as members shared experiences about the LF treatment and how people felt afterwards.

Although these findings are situated within a research setting, there are implications for routine MDA programs, regardless of the drug regimen used. First, a purposeful approach to AE management and communication must be included in MDA programs. Specific messages of importance include where help can be accessed (e.g. by phone or in person) and free access to medicines (if needed) will reassure community members and potentially reduce the fear people may have about taking the LF treatment. Secondly, some of the elements of professionalism highlighted in the qualitative data can be applied to routine MDA settings without significant cost implications. Specifically, information provision prior to distribution of treatment and allocating sufficient time for questions from community members. The provision of directly observed therapy was also appreciated and demonstrated that those distributing the treatment cared about community members. These improvements in the quality of delivery of MDA can attract individuals who have never taken LF treatment previously and can complement the delivery of IDA as a new treatment regimen. Regardless of the drug regimen used, delivering a quality MDA remains essential.

Finally, this study has broken new ground by embedding acceptability assessments into a study of a new NTD intervention. Acceptability incorporates themes that extend beyond coverage, the most commonly used measure of MDA success. The mean acceptability score combined perception of efficacy of treatment, relevance to community and personal preferences and needs, attitudes towards the treatment and intention to participate again. This measure allowed for a more nuanced understanding of what factors might be associated with uptake or compliance and what might be associated with behavior in future rounds. In light of documented coverage–compliance gaps [42], measuring coverage alone may not be a sufficient indicator of MDA success. Acceptability studies provide important additional information, assessing the impact of information campaigns, participant experiences and attitudes toward the intervention that may affect its success. Indicators of acceptability can be considered as complementary indicators to coverage and compliance. Components of acceptability can be included in the design of MDA strategies including social mobilization, community engagement and training of personnel to ensure that important gaps are addressed.

### Limitations

Study site was the strongest predictor of acceptability in this study and may have reflected some of the variability of approaches towards social mobilization and education about the safety trial by the safety trial teams. In Fiji, where the highest mean acceptability score was reported, some individuals reported near perfect scores across all the 9 indicators. There are some possible reasons to explain the high scores in Fiji. The safety trial in Rotuma and Gau was preceded with a comprehensive promotional campaign that reached the population widely focusing on LF but also the off-target benefits on scabies and soil-transmitted helminths that are both endemic in Fiji. The promotion of benefits of treatment on the three NTDs may have had an important effect on acceptability in Fiji, where scabies is more prevalent than reported in the other sites [43].

The second limitation of this research is that there are no historical data with which to compare the mean acceptability scores generated here. However, there is strong support for the research methodology of concurrent qualitative and quantitative data collections to ensure validity across the findings. In addition, the measure of acceptability was built upon a previously developed scale and was adjusted using validated questions used in LF MDA studies [44–46]. Results from the mean acceptability scores were further validated through the qualitative research collected concurrently by the research team. This paper demonstrates the utility of this new measure to combine perceptions, knowledge, intention for future uptake and relevance of the intervention. These variables when taken independently do not offer the same weight as their combined score. Finally, because the study sample was taken from individuals who ingested the treatment during the safety trial, we are not able to compare acceptability scores between those who took the treatment and those who did not. Future research can build upon this data in larger community rollouts of MDA.

## Summary and conclusions

- The new triple drug (IDA) and the standard two drug (DA) MDA regimens for LF elimination were highly acceptable and equally acceptable in communities that participated in the community-based safety trial in Fiji, Haiti, India, Indonesia, and Papua New Guinea.
- The mixed model analyses revealed significant variability between countries in aggregate acceptability, but there were no significant differences between the two treatment groups (IDA vs DA) overall or in any single country.
- Adverse events, presence of microfilaremia or filarial antigenemia were not associated with acceptability.
- Although both males and females had high levels of acceptability, women had lower mean acceptability scores then men. In addition, females had higher reported clinical and perceived AE following treatment. Further understanding of the differences between men and women with regards to AE and acceptability, and their potential interaction are needed.
- Increased number of pills consumed was acceptable when accompanied with appropriate health messaging about their action.
- High levels of professionalism were appreciated by study participants, and this increased uptake of LF treatment by individuals who had never previously complied with MDA.
- Although the community-based safety trial involved a professional health workforce and had more resources than a regular MDA, lessons learnt about social mobilization, timely communication, use of directly observed therapy, reassurance about the management of adverse events and explanations about number of pills can be applied to any MDA using either drug regimen with minimal or no additional cost.
- Communities where scabies occurred appreciated the added value of ivermectin for reducing itching. This has implications for the promotion of IDA and can encourage community members to participate in MDA with the new treatment.
- Where STH were common, community members appreciated the added value of the MDA to reduce worm burdens.
- Many members of communities that have had multiple prior rounds of MDA still lack knowledge and understanding about the role of treatment for preventing LF. This may be because they were young at the time MDA began or because they cease to see evidence of LF infection in their communities. Regardless of the treatment regimen to be employed, special attention should be given to developing tailored messages that consider sex and age in MDA promotion activities.

## Supporting information

S1 Protocol Global acceptability protocol. (PDF)

S1 Table Summary of sociodemographic and socioeconomic variables for all five countries. (XLSX)

S2 Table Results from mixed-model analysis of aggregate acceptability scores. (XLSX)

S3 Table Plots of participant response to acceptability subscore questions stratified by country. (XLSX)
