# Supplementary material for: A multicenter, community-based, mixed methods assessment of the acceptability of a triple drug regimen for elimination of lymphatic filariasis
Source: PLoS Negl Trop Dis. 2021 Mar 3;15(3):e0009002. doi: 10.1371/journal.pntd.0009002 (PMC7928496; doi:10.1371/journal.pntd.0009002)
Supplement: S1 Protocol — (PDF) [file pntd.0009002.s001.pdf]

“Treatment acceptability study following the Community Based Safety Study of 2-drug (Diethylcarbamazine and Albendazole) versus 3-drug (Ivermectin, Diethylcarbamazine and Albendazole) Therapy for Lymphatic Filariasis in Fiji, Haiti, India, Indonesia and Papua New Guinea”

Type: Survey to understand community acceptability of LF drugs

Financing: This project is financed by the Bill and Melinda Gates Foundation through the Death for Onchocerciasis and Lymphatic Filariasis (DOLF) Project at the University of Washington, USA and the NTD Support Center at the Task Force for Global Health, USA

Project duration: July 2016 – July 2017

Date of protocol version: 8 November 2016, addition of updated final questionnaire

DOLF Project Principal Investigators:
Gary Weil, MD, Washington University, USA
Christopher King, MD, PhD, MPH, Case Western Reserve University, USA

Principal Investigator for acceptability study:
Alison Krentel, PhD, MScPH, Bruyère Research Institute, Canada

Study Site Principal Investigators:
(Fiji) Myra Hardy MD, University of Melbourne, Australia and Josaiah Samuela, Ministry of Health and Medical Services Fiji

(Haiti) Jean Frantz Lemoine, MD, MPH, Coordinator of National LF and Malaria Programmes, Ministry of Health, Haiti

(Indonesia) Taniawati Supali PhD, Universitas Indonesia, Indonesia

(India) P. Jambulingam, MD and Vijesh Sreedhar Kuttiatt, MD, Vector Control Research Centre, Indian Council of Medical Research, India

(Papua New Guinea) Leanne Robinson, PhD, MPH and Peter Siba, PhD, Papua New Guinea Institute for Medical Research, Papua New Guinea

Table of Contents

PRINCIPAL ROLES **4**
DOLF Project Principal Investigators 4
Principal Investigator for the acceptability study 4
Study Site Principal Investigators 4
Co-Investigators 5
Fiji 5
Haiti 6
India 6
Indonesia 6
Papua New Guinea 7
Protocol Statistician 7

1. SUMMARY OF THE PROTOCOL **8**

2. RATIONALE FOR THE STUDY **9**

3. STUDY OBJECTIVES **11**

4. COMMUNITY SURVEY **11**
4.1. Timing 11
4.2. Questionnaire development 11
4.3. Sampling frame 12
4.4. Analysis 14

5. FOCUS GROUP DISCUSSION **15**
5.1. Timing 15
5.2. Sampling frame 15
5.3. Development of the topic guide 15
5.4. Analysis of the FGD 16

6. IN DEPTH INTERVIEWS WITH KEY INFORMANTS **16**
6.1. Timing 16
6.2. Sampling frame 16
6.3. Development of the topic guide 16
6.4. Analysis of the IDIs 17

7. ETHICAL CONSIDERATIONS **17**
7.1. Community survey 17
7.2. Focus Group discussions 17
7.3. In depth interviews with key informants 18

8. SAMPLE TIMELINE **19**

REFERENCES **20**

APPENDIX 1 INFORMATION SHEET AND INFORMED CONSENT FOR COMMUNITY SURVEY **22**

APPENDIX 2 INFORMATION SHEET AND INFORMED CONSENT FOR IN DEPTH INTERVIEWS WITH KEY INFORMANT **24**

APPENDIX 3 INFORMATION SHEET AND INFORMED CONSENT FOR FOCUS GROUP DISCUSSION PARTICIPANTS **26**

APPENDIX 4 COMMUNITY QUESTIONNAIRE **28**

APPENDIX 5 TOPIC GUIDE FOR IN DEPTH INTERVIEWS **39**

APPENDIX 6 TOPIC GUIDE FOR FOCUS GROUP DISCUSSIONS **43**

APPENDIX 7 TOPIC GUIDE FOR FOCUS GROUP DISCUSSION IN TREATMENT NAÏVE AREAS **48**

Principal Roles

DOLF Project Principal Investigators

Gary Weil, MD
Department of Medicine
Washington University School of Medicine
444 Forest Park Ave
St. Louis, MO, 63110 USA
Email: gary.j.weil@wustl.edu

Christopher King, MD, PhD, MPH, Case Western Reserve University, USA
Center for Global Health and Diseases
Biomedical Research Building
2109 Adelbert Rd.
Case Western Reserve University School of Medicine
Cleveland, OH, 44106-4983
Tel: 216-368-4817,
Email: cxk21@case.edu

Principal Investigator for the acceptability study

Alison Krentel PhD, MScPH
Bruyère Research Institute
85 Primrose Avenue, room 308-B
Ottawa, ON K1R 6M1, Canada
Tel: +1(613) 562-6262 x2954
Email: akrentel@yahoo.ca

Study Site Principal Investigators

Andrew Steer MD, PhD
Department of General Medicine, Royal Children's Hospital
Centre for International Child Health, University of Melbourne
Melbourne, Australia
email: andrew.steer@rch.org.au

Josaiah Samuela
Ministry of Health and Medical Services Fiji
Suva, Fiji
Email : josaia.samuela@health.gov.fj

Jean Frantz Lemoine, MD, MPH
Programme National de la Malaria et de la Filariose Lymphatique (PNCM)
Ministère de la Santé Publique et de la Population (MSPP)
Rue Monseigneur Guilloux, Palais des Ministères, Port-au-Prince, Haïti
Tel: +1 (509) 3744-8755 or +1 (509) 3946-3026
Email: tileum@hotmail.com

Dr. P. Jambulingam
Scientist “G” and Director
Vector Control Research Centre
Indira Nagar, Medical Complex
Pondicherry 605 006, India
Tel: +91 413 227 2422
Email: pcsaja@yahoo.co.uk

Dr. Vijesh Sreedhar Kuttiatt
Scientist “E”
Vector Control Research Centre
Indira Nagar, Medical Complex
Pondicherry 605 006, India

Taniawati Supali, PhD
Department of Parasitology
Universitas Indonesia
Tel: +62 812 8400 5628
Email: taniawati@yahoo.com

Leanne Robinson, PhD, MPH
Papua New Guinea Institute of Medical Research (PNG IMR)
Madang, PNG
Tel: 675-422-2909
Email: robinson@wehi.EDU.AU

Peter Siba, PhD
Papua New Guinea Institute of Medical Research (PNG IMR)
Goroka, PNG
Tel: 675-532-2800
Email: peter.siba@pngimr.org.pg

Co-Investigators

Fiji

Myra Hardy MD
University of Melbourne
Melbourne, Australia
Email: Myra.Hardy@rch.org.au

John Kaldor, MD, PhD
Public Health Interventions Research Group
Kirby Institute, University of New South Wales
Sydney, Australia
Tel: +61 (0)2 9385 0900
email: jkaldor@kirby.unsw.edu.au

Lucia Romani, PhD
Kirby Institute, University of New South Wales
Sydney, Australia
email: lromani@kirby.unsw.edu.au

Margot Whitfeld, MD
Department of Dermatology
St. Vincent's Hospital
Sydney, Australia
email: mwhitfeld@stlderm.com.au

Haiti

Christine Dubray, MD, MSc
Division of Parasitic Diseases and Malaria, CDC
1600 Clifton Road, MS A-06
Atlanta, GA 30317, USA
Tel: +1(404)718-4769
email: ffg5@cdc.gov

Valery Madsen Beau de Rochars, MD, MPH
Emerging Pathogens Institute, University of Florida
Tel: +1(352) 294-5695
Fax: +1 (352) 273-9430
email: madsenbeau@phhp.ufl.edu

Abdel N. Direny, MD, MPH
RTI Envision
Washington DC, USA
Tel: +1 (202) 974-7814
Email: adireny@rti.org

Carl Renand Fayette, MD
IMA World Health
Port au Prince. Haiti
email: carlfayette@imaworldhealth.org

India

Ms. B. Nandha
Scientist "B"
Vector Control Research Centre
Pondicherry, India
Email: nandabasker@gmail.com

Indonesia

Michael Christian MD
Department of Parasitology
Universitas Indonesia
Jakarta, Indonesia

Tel: +62812 3758 7120
Email: michael.christian9591@gmail.com

Maddi Djara, Mataram, Indonesia
Email :maddidjara@gmail.com

Peter Fischer, PhD
Washington University School of Medicine
Infectious Diseases Division
444 Forest Parkway, Room 4182A
St. Louis, MO 63110 USA
Tel: +1(314) 454-7876
Email: Pufische@dm.wustl.edu

Adriani Lomi Ga, ST. Planning Department in East Nusa Tenggara Provincial Government, Kupang, Indonesia
Email : anilomiga@gmail.com

Papua New Guinea

Moses Laman, MBBS, PhD email: drmlaman@yahoo.com
Livingstone Tavul, MSc email: ltteruah1@gmail.com
PNG IMR
Madang, PNG
Tel: 675-852-2909

James Kazura, MD, Tel 216-368-4810; email: jxk14@case.edu
Daniel Tisch, PhD, email: dxt37@case.edu
Cade Howard, email: sxh817@case.edu
Krufinta Bun, email: krufinta.bun@case.edu
Center for Global Health and Diseases
Biomedical Research Building
2109 Adelbert Rd.
Case Western Reserve University School of Medicine
Cleveland, OH, 44106-4983

Protocol Statistician

Ken Schechtman, PhD,
Dept. of Internal Medicine and Division of Biostatistics
Washington University School of Medicine
660 South Euclid, St. Louis, MO 63110
Tel: +1(314)362-2271
Email: kschechtman@wustl.edu

1. Summary of the protocol

**Title of the study:**	Treatment acceptability study following the Community Based Safety Study of 2-drug (Diethylcarbamazine and Albendazole) versus 3-drug (Ivermectin, Diethylcarbamazine and Albendazole) Therapy for Lymphatic Filariasis in Papua New Guinea
**Study Type:**	Survey to understand community acceptability of LF drugs
**Population:**	Participants aged 14 and over who received 3 drugs (IDA) for the community study<br><br>Participants aged 14 and older who received 2 drugs (DA) for the community study
**Number of sites included in the study:**	Study sites will be communities in:<br><br>Fiji: communities in Gau Island, with possibility of Rotuma Island, if alternative site is needed<br><br>Haiti: Commune of Quartier Morin<br><br>India: Selected communities in Yadgir district, Karnataka<br><br>Indonesia: Kahale and Karang Inda in Sumba Barat Daya district, Nusa Tenggara Timur Province<br><br>Papua New Guinea: Madang Province, Bogia District and as alternatives, Dreikiker District, East Sepik Province and East New Britain Province
**Duration of Study Participation:**	<2 hours
**Primary objective:**	Assess the overall acceptability of the 3-drug treatment in the community as compared to the 2-drug treatment
**Secondary objectives:**	1. Measure the perception of adverse events (AE) reported by safety trial participants, comparing those in the 2-drug versus 3-drug arms<br>2. Assess the overall acceptability in the community of the 3-drug regimen, as compared to the 2-drug regimen<br>3. Assess the overall acceptability in the community of those individuals who are (microfilaria) mf positive, as compared

	to those who are mf negative<br>4. Investigate the acceptability and feasibility of delivering the 3-drug regimen
**Incorporation into the DOLF project**	Data will be available and reviewed at a country level and at the project level. This protocol outlines the collection and analysis of the aggregated data from all sites,

As part of the larger “Community Based Safety Study of 2-drug versus 3-drug Therapy for Lymphatic Filariasis” a study to assess treatment acceptability in the community is planned in each research site: Fiji, Haiti, India, Indonesia and Papua New Guinea. The overall aim of this research is to understand the community’s acceptance of the 3-drug regimen as well as gain insight into the feasibility of administering this new therapy in the future. Community acceptance will be measured using a survey to community members receiving treatment during the trial. Part of the investigation will include assessing community member’s perception of the possible adverse events experienced as a result of the 3-drug therapy, and how that might affect future rounds of mass drug administration (MDA) at the community level. In addition, focus group discussions (FGD) will be carried out with community members and community health workers to further investigate acceptability of the new therapy. To complement the community survey and focus group discussions, a series of key informant interviews are proposed with community leaders and health personnel in the same communities to assess perceptions about the 3-drug *versus* the 2-drug regimen as well as gain insight into the feasibility of distributing the new regimen as well as perceptions about managing adverse events.

2. Rationale for the study

With the introduction of a new treatment regimen for the elimination of lymphatic filariasis (LF), understanding community perceptions about the treatment, its adverse events (AE) as well as its efficacy will be an important component of assessing the acceptability of the 3-drug therapy. In particular, perceptions about the severity of experienced or observed AE, the efficacy of the treatment in killing the worms and understanding the positive presence of AE will be important to investigate.

Research has demonstrated the important impact that AE can have on individuals’ acceptance of LF treatment using the 2-drug regimen [1, 2]. In some areas where MDA has been ongoing for many years, we might expect these AE to be objectively of minimal clinical significance, yet subjectively community members continue to report “fear of AE” as a deterrent to comply with MDA. In recent research in a low prevalence area in Indonesia, 33% of individuals interviewed reported experiencing some form of side effect or AE as a result of taking the LF treatment (A. Krentel personal experience). Thomsen et al (2016) reported a higher rate of AE in those who were administered the 3-drug regimen versus those who received the 2-drug therapy [3]. As the wider application of this new therapy is considered, it will be important to understand if the perception of these AE is different in between the two treatment arms.

Another important deterrent to compliance with MDA is a lack of understanding of the benefit of treatment [4, 5]. The 3-drug regimen has been shown to be highly effective in the reduction of microfilariae [3]; therefore communicating this message to participants will be of crucial importance. Measuring participants' understanding of this message will be essential in determining their acceptance of AE associated with the treatment. In PNG and in neighboring Indonesia when communities understand the reasons AE occur, they welcome them as a sign that the drugs are working [6, 7]. Knowing if this message also works with the 3-drug therapy where more AE are expected to occur is important in the future promotion of this treatment.

For the purposes of this research, a mixed method approach is recommended, combining the use of a community survey, focus group discussions and in depth interviews with key informants. The community survey will allow a robust comparison of treatment acceptability between those receiving the 2-drug regimen and those receiving the 3-drug regimen. A composite score will measure acceptability, combining outcomes like the respondents' intention to take the treatment again and willingness to recommend it to other family members. Acceptability will be analyzed by the impact of some of the known factors that impact compliance: perception of AE, knowledge about AE, perceptions about the drug characteristics (safe, number of pills, taste), knowledge of vector, belief that the treatment is associated with health, and others. In order to assess the difference between the two treatment arms, the sampling frame for the community survey will take into account which regimen the individual received.

To complement the community surveys and provide further in depth analysis, focus group discussions (FGD) are planned with specific groups in the community, namely men, women, young people and community health workers. The FGDs will provide further insight and depth for some of the questions asked in the community survey. Specifically FGDs will investigate issues expected to relate to the 3-drug regimen: number of pills, perception of AE, how to ensure directly observed treatment and proposed messages to encourage compliance.

These results will be further substantiated by interviews with key community leaders, as well as community and professional health workers working in LF elimination at the village level. These interviews will provide an understanding of the macro level issues that key informants perceive as critical to consider with the use of the 3-drug therapy. With this, interview respondents will be asked what advantages and concerns they have with regards to the 3-drug regimen based on their participation in and understanding of the safety trial.

The outcome of this research will provide operational recommendations to accompany the safety study. These will inform additional acceptability research if the 3-drug regimen is adopted as global policy. An important outcome will be to determine if there are any real differences in community acceptance of the 3-drug regimen when compared to the standard treatment. If there are any differences, then further investigation may be recommended. In addition, the global programme will need to consider how to adjust the delivery protocols and recommended messages used by community drug distributors giving out the 3-drug regimen. The acceptability study will provide a preliminary understanding of these issues and will provide important insights into the use of this regimen on a wider scale.

3. Study objectives

1) Measure the perception of AE reported by safety trial participants, comparing those in the 2-drug *versus* 3-drug arms
2) Assess the overall acceptability in the community of the 3-drug regimen, as compared to the 2-drug regimen
3) Assess the overall acceptability in the community of those individuals who are mf positive, as compared to those who are mf negative
4) Investigate the acceptability and feasibility of delivering the 3-drug regimen

4. Community survey

Community surveys are often called Knowledge, Attitudes and Practice (KAP) surveys because they use a cross sectional survey design to understand what community members *know* about disease, treatment and prevention; how they *perceive* factors related to the disease and finally what they *do* about it (e.g. take a drug, hang a bednet, use a condom). For the purposes of this survey, it is recommended to use a cross sectional survey design. However the terminology and format of the KAP may not be the most appropriate questionnaire design for the study proposed. Specific knowledge about LF disease is not a strong predictor for compliance in MDA for LF, with the exception of knowing that mosquitoes transmit LF [8, 9]. For the purposes of this research, focusing on knowledge of LF disease may not inform community acceptability of the 3-drug regimen as compared to the 2-drug regimen. Furthermore research has shown that there are important intrinsic reasons that affect people's decisions to take or not to take the LF treatment during MDA. Social norms of compliance, emotional cues, altruism and an individual's personal situation have all been shown to be associated with taking the LF drug [5, 10-12]. Understanding some of these intrinsic factors associated with taking the 3-drug regimen as opposed to the 2-drug regimen will be important in building a picture of community acceptability. As a result, although there may be similarities in some of the questions asked, it is recommended to call the community survey a "treatment acceptability survey" as opposed to a "KAP survey."

4.1. Timing

In order to allow time between the clinical assessment and monitoring of AE in the community trial as well as some time for the effects of ivermectin to become apparent, individuals will be approached for enrollment in the community acceptability survey within four months after their enrollment in the safety trial. As the safety trial will be a memorable event in the community, it is expected that the effect of recall bias will be minimal among respondents [13].

4.2. Questionnaire development

Questionnaire development is based on previous LF surveys carried out in Indonesia and in Papua New Guinea. In addition, known influences based on the most recent literature on compliance will be included in the acceptability survey, where appropriate. Validated questions from acceptability research as well as quality of life indicators for scabies will be included in the questionnaire.

Questionnaires will be written in English and translated into the local language. In order to test the understandability of the questionnaire with the local population, the enumerators will give advice on the vocabulary used during the training and a small sample of individuals will be administered the questionnaire prior to survey implementation. At the end of this testing, these respondents will be asked to comment on the questions themselves, whether they were clear and the language was appropriate. Changes will be made if needed. The questionnaire will then be translated back into English.

4.3. Sampling frame

In estimating the sample size for the acceptability survey, one of the challenges we have is that we do not know the estimated acceptability rates in people who have received the 3-drug regimen. From recent research in Indonesia (A. Krentel, personal experience) in low (mf rate=1%) and high prevalence (mf=8%) areas, we know that acceptability with DEC+ALB, as measured in the intent to take the LF drugs again, was measured as 79% and 82% respectively.

Because we do not have a 3-drug acceptability rate, we cannot estimate the difference we might expect in between the regimen groups. As a result, this survey will create preliminary data, estimating the difference in acceptability rates between those individuals receiving the 2 and 3 drug regimens as well as the difference in rates between those with positive mf rates at the start of the safety trial and those who are mf negative. This survey will provide insight into possible trends in acceptability and will inform if further investigation is needed.

The range of mf prevalence is expected to be wide in between the study sites. As such, we will oversample those areas where it will be higher. The target samples for the acceptability survey are as follows:

- 400 individuals in Papua New Guinea
- 400 individuals in Indonesia
- 400 individuals in Haiti
- 450 individuals in Fiji
- 400 individuals in India

Within each country, equal numbers of subjects will be enrolled in the two drug and the three drug intervention arms. In PNG, we will oversample those villages were LF prevalence is expected to be higher. With a projected 50% infection rate, this is the only participating country that is expected to have an infection rate in excess of 10%. It is therefore the only country that will provide a universe of infected subjects that is sufficiently large to provide accurate measures of the acceptability of study medications in subjects who are infected. Specifically, if we add the 50 subjects who are expected to be infected in Indonesia, Haiti, and Fiji combined to the 200 anticipated infected subjects in PNG, we anticipate that this acceptability study will enroll 250 infected subjects, or 125 in each intervention arm. With a total of 125 infected subjects in each arm, a dichotomous measure (yes or no) of the acceptability of a particular drug combination will, with 95% certainty, differ by no more than 9% from the true acceptability rate.

In Fiji, as there will be three study arms, we expect to enroll 150 individuals per treatment arm. For the aggregated analysis, we will include 300 individuals in total, 150 for those receiving the 3-drug regimen once and those receiving the 2-drug regimen once, as is the protocol in the other countries. The data from the total 450 participants will be interpreted and analyzed at the country level.

Because we expect the acceptability of a particular drug combination to be highly correlated within families, our protocol permits the enrollment of precisely one subject from each participating family. Eligible family members will have to be at least 14 years of age. As chronic manifestations of the disease begin to show at adolescence, so personal experience with LF may also begin at this age [14]. Both men and women will be included in the sample.

Enrollment procedures are as follows. Our goal will be to interview acceptability study participants beginning at least two weeks after the AE monitoring has been completed within a given village. The reason for waiting at least two weeks is that we want to minimize the likelihood that we will interview individuals who are still experiencing the symptoms of any adverse events that may have occurred in the parent study. If we were to conduct acceptability interviews while subjects were still experiencing adverse effect symptoms, the immediacy of those symptoms might bias subjects' perceptions of acceptability.

All families within a village that participated in the parent safety study will be eligible to participate in this acceptability study, with consent for such participation having been a part of the consent form of the parent study. Within each country, we will enter villages sequentially in the order that they were entered in the parent study. We will continue to interview subjects in a particular country until the target number within a given study arm has been reached. When the target number has been reached in one study arm, we will stop enrolling subjects in that arm and will continue enrolling in the other study arm until the target has been reached.

The family member to be interviewed for this study will be selected using the following procedures. A list of family members over the age of 14 who have been enrolled in the parent study will be compiled as soon as possible after the parent study has been concluded in a village. From this list, a random family member will be selected as the target participant in the acceptability study. When we approach a household, we will seek to interview the randomly selected family member. If that family member is not immediately available, we will discuss whether the logistics of conducting the interview with that family member at a different time are feasible. If we conclude that waiting for that alternative time is not reasonable or if the selected family member does not wish to participate, we will conduct the interview in whatever family member is available, independent of which individual has been randomly selected.

Enumerators will travel to the house to interview the identified individual. Data will be collected using the REDCap system.

4.4. Analysis

Outcome of interest:

Acceptability of the 3-drug therapy will be measured in a composite score from the following questions[1]:

- Intention to take LF drugs in the future measured on a 5-point scale ranging from "I will never take this drug again" to "I will definitely take this drug again." (Adapted from Liau and Zimet 2001)
- Willingness to encourage other family members to take the LF drug, if offered in the future measured as a 5-point scale ranging from "I will never encourage my family to take the LF drugs" to "I will definitely encourage my family to take the LF drugs."
- Overall feeling about the LF elimination program as a 5-point scale ranging from "Very negative" to "Very positive"
- Perception of health since taking the LF drugs as a 5-point scale ranging from "Considerably worse" to "greatly improved"
- In addition to the scoring, each outcome can be converted to a binary variable for multivariate modeling.

Inputs / Exposure variables:

- SES data
- Data from safety trial (clinical presence of AE, mf rate, household information)
- Treatment arm (2-drug versus 3-drug)
- Informed about the treatment before receiving the drug (e.g. did they receive any information)
- Belief in the efficacy of the treatment to eliminate / prevent LF (e.g. believe that the drugs work to prevent / treat LF)
- Belief in the efficacy of the treatment to treat scabies (e.g. believe that the drugs work to treat scabies)
- Belief in the efficacy of the treatment to treat other intestinal worms (e.g. believe that the drugs work to treat worms)
- Knowledge of the 'positive' component of AE (e.g. occur because the medicine is working)
- Perception of AE (e.g. none, mild, moderate, severe)
- Understanding that taking LF medicine is good for promoting health
- Knowledge that mosquitoes transmit LF
- Perception that the rest of the family/ household would take the LF drugs, if offered in the future (yes/no)
- Belief that the drug distributors are doing a good job (using a 10-point scale)
- Perceptions of the drugs (e.g. safe, neutral, dangerous)
- Components of the drugs (e.g. number, size, taste of pills)
- Emotions surrounding LF treatment (e.g. how does taking LF treatment make you feel?)

Analysis procedures

For the data cleaning and data reduction, the following steps will be performed:

---

[1] Scoring concept amended from Treatment Acceptability Research (see Carter SL (2007) "Review of Recent Treatment Acceptability Research" *Education and Training in Developmental Disabilities* 42(3): 301-316.

- Check response bias
- Clean the raw data set (range check, consistency checks)
- Transfer corrected data set to STATA statistical software (Stata Corporation, College Station, Texas).
- Group continuous variables into categorical variables, namely age. Recode certain variables where needed.

For the analysis, a descriptive analysis of the whole dataset will be prepared. The data from the community survey will be linked to the safety trial within the REDCap system.

Likert scales will be analyzed as both dichotomous and as continuous variables.

For both of the predictors of acceptability (drug regimen and presence of mf) logistic regression models will be created. Presence of AE as measured in the clinical surveys will be considered in the analysis, as will subjective perceptions of AE.

5. Focus Group Discussion

5.1. Timing

The focus group discussions will take place at the same time as the community survey, in the same communities.

5.2. Sampling frame

For the focus group discussions, we will identify persons from specific groups of people: women of reproductive age, young people, men and community health workers. The rationale behind the selection of each of these groups is related to the prevailing evidence of their participation in MDA in the literature. Women of reproductive age often do not comply with treatment because they are either pregnant or breastfeeding, however they are often the gatekeepers for health in the household and ensure members of their household take the treatment when offered. Men and young people have been known to be less compliant with MDA and so understanding their perceptions about the 3-drug regimen, MDA in general and soliciting their advice about how best to promote and reach their communities will be informative. Finally, as community health workers are usually the persons responsible for distributing the drug at the community level, understanding their perspectives on DOT, AE and messaging for the 3-drug regimen is important.

For the FGD, women, young men and men will be selected from the cohort of individuals receiving the 3-drug regimen.

5.3. Development of the topic guide

<u>Range of issues to explore include:</u>

- How is LF elimination different / similar from the other health programs in their village?
- What are the health benefits from taking the treatment?
- What are the social benefits from taking the treatment?

- Do people like to take the pills in front of the distributor? Why or why not?
- How do you feel about the number of pills that you have to take?
- Why don't people want to take it?
- Did you have any side effects after you took the drugs (positive or negative)? How did you feel about them?
- What suggestions do you have to promote MDA to their community? Household?
- Are there any specific messages you would recommend to us?

5.4. Analysis of the FGD

Recorded focus group discussions will be transcribed word for word in the local language. They will be translated into English. A second researcher with knowledge of English and the local language will check translation, sampling portions of each transcript and back translating them from English to the local language to check the reliability of the translation. The researchers will read through each transcript, recording emergent themes in an Excel matrix. NVivo will be used to assess trends and patterns in the interview transcripts.

6. In depth interviews with key informants

6.1. Timing

The key informant interviews will take place at the same time as the community survey, in the same communities.

6.2. Sampling frame

A purposive sampling frame will be used, with individuals identified based on their leadership and cultural position with the village as well as their involvement with LF elimination and with the community trial. With this in mind, a range of 8-10 individuals will be included in the sample. In order to understand the acceptability of administering the 3-drug regimen, individuals to be interviewed would need to be those persons who are either directly involved with LF activities in the village or who *would be* involved in MDA in the future. Suggestions include community and/or religious leaders, community health workers, teachers.

6.3. Development of the topic guide

<u>Range of issues to explore include:</u>

- What are the advantages of the 3-drug therapy in MDA? Disadvantages?
- What opportunities do they see in the administration of the 3-drug therapy, versus the 2-drug therapy?
- What concerns or challenges do they see in the administration of the 3-drug therapy, versus the 2-drug therapy?
- How do they feel about the number of pills that the community is asked to take?
- How do they feel about the side effects people might have / have?
- What suggestions do they have to promote MDA in this village? This province? The country? What messages would they recommend using?
- Which groups of people do they think will be difficult to reach with future MDA? Why? Any advice to approach them?

6.4. Analysis of the IDIs

Recorded interviews will be transcribed word for word in the local language. They will be translated into English. A second researcher with knowledge of English and the local language will check translation, sampling portions of each transcript and back translating them from English to the local language to check the reliability of the translation. The researcher will read through each transcript, recording emergent themes in an Excel matrix. NVivo will be used to assess trends and patterns in the interview transcripts.

7. Ethical Considerations

In the parent safety trial informed consent form in each country, the participant is notified that they may be contacted for a follow-up survey so each participant will have agreed to be identified for the purposes of the acceptability study during the informed consent process for the safety trial.

7.1. Community survey

Ethical approval will be obtained from the local national research institution in each country as well as Washington University in St. Louis, Case Western Reserve University, University of Melbourne and Bruyère Research Institute.

Prior to giving consent to participate, the enumerator will read out the information sheet in the local language containing the aim of the survey, the length of time it is expected to take (15 minutes) as well as the protection of confidentiality for each respondent. Following this, each respondent will be asked to sign the informed consent form and where respondents are illiterate, a mark can be made. The enumerator will indicate that informed consent has been given. Age of eligible respondents is 14 years of age and older. For those aged 14 – 18 years, parental consent will be sought and provided on the informed consent form before the interview can begin. All forms will remain with the research team and will not contain any personal information other than the individual's signature.

At the end of the interview, each respondent will be given an information sheet with the principal investigator's contact details, should there be any questions. With this sheet, the respondent will also receive a brief information sheet on lymphatic filariasis, the mass drug administration and who is eligible for treatment.

The data will be stored on Washington University servers during the duration of the study. After the study ends, electronic copies of the de-identified datasets will be kept by the PI indefinitely.

7.2. Focus Group discussions

Ethical approval will be obtained from the local national research institution in each country as well as Washington University in St. Louis, Case Western Reserve University, University of Melbourne and Bruyère Research Institute.

The interviewer will read the informed consent form to each person participating in the focus group discussion. The respondents will be asked to each sign an informed consent form for their participation. All interviews will be recorded with the permission of the respondent. Where permission is not granted, the interviewer will ask to take notes throughout the interview.

Any identifying information (name, address) will not be recorded. Individuals will not be identified in the transcripts or in the recordings and their anonymity will be maintained in all reporting and in the manuscripts. Transcripts of the interviews will remain with the research team.

The data will be stored with the PI, under password protection. After the study ends, electronic copies of the de-identified datasets will be kept by the PI indefinitely.

7.3. In depth interviews with key informants

Ethical approval will be obtained from the local national research institution in each country as well as Washington University in St. Louis, Case Western Reserve University, University of Melbourne, and Bruyère Research Institute.

The interviewer will read the informed consent form to each person participating in the interview. The respondents will be asked to sign an informed consent form for their participation. All interviews will be recorded with the permission of the respondent. Where permission is not granted, the interviewer will ask to take notes throughout the interview.

Any identifying information (name, address) will not be recorded and the identity of the respondent will be kept confidential in reporting. Transcripts of the interviews will remain with the research team. The data will be stored with the PI under password protection. After the study ends, electronic copies of the de-identified datasets will be kept by the PI indefinitely.

8. Sample timeline

Activities	May 2016				Jun 2016				July 2016				Aug 2016				Sept 2016				Oct 2016				Nov 2016				Dec 2016				Jan 2017				Feb 2017			
	S1	S2	S3	S4	S1	S2	S3	S4	S1	S2	S3	S4	S1	S2	S3	S4	S1	S2	S3	S4	S1	S2	S3	S4	S1	S2	S3	S4	S1	S2	S3	S4	S1	S2	S3	S4	S1	S2	S3	S4
**Planning for study**																																								
Study planning																																								
Research assistant recruited																																								
Ethical approval																																								
Set up the activities																																								
Finalise the study questionnaires (translation and language comprehension																																								
Prepare training guide for interviewers																																								
Recruit and train enumerators for the study																																								
**Data collection**																																								
Carry out the community survey																																								
In depth interviews and FGD																																								
Transcription & translation																																								
**Analysis and dissemination**																																								
Data analysis																																								
Report writing																																								
**Community drug distribution (parent study)**																																								

References

1. Krentel A, Fischer PU, Weil GJ. A Review of Factors That Influence Individual Compliance with Mass Drug Administration for Elimination of Lymphatic Filariasis. PLoS neglected tropical diseases. 2013;7(11):e2447. Epub November 21, 2013. doi: doi:10.1371/journal.pntd.0002447.
2. Babu BV, Babu GR. Coverage of, and compliance with, mass drug administration under the programme to eliminate lymphatic filariasis in India: a systematic review. Transactions of the Royal Society of Tropical Medicine and Hygiene. 2014;108(9):538-49. Epub 2014/04/15. doi: 10.1093/trstmh/tru057. PubMed PMID: 24728444.
3. Thomsen E, Sanuku N, Baea M, Satofan S, Maki E, Lombore B, et al. Efficacy, Safety, and Pharmacokinetics of Coadministered Diethylcarbamazine, Albendazole, and Ivermectin for Treatment of Bancroftian Filariasis. Clinical Infectious Diseases. 2016;62(3):334-41. doi: 10.1093/cid/civ882.
4. Ramaiah KD, Kumar KNV, Hosein E, Krishnamoorthy P, Augustin DJ, Snehalatha KS, et al. A campaign of 'communication for behavioural impact' to improve mass drug administrations against lymphatic filariasis: structure, implementation and impact on people's knowledge and treatment coverage. Annals of Tropical Medicine and Parasitology. 2006;100(4):345-61. doi: 10.1179/136485906x105598. PubMed PMID: WOS:000238656500007.
5. Babu BV, Suchismita M. Mass drug administration under the programme to eliminate lymphatic filariasis in Orissa, India: a mixed-methods study to identify factors associated with compliance and non-compliance. Transactions of the Royal Society of Tropical Medicine and Hygiene. 2008;102(12):1207-13. doi: http://dx.doi.org/10.1016/j.trstmh.2008.05.023. PubMed PMID: 20083317171.
6. Bockarie MJ, Tisch DJ, Kastens W, Alexander NDE, Dimber Z, Bockarie F, et al. Mass treatment to eliminate filariasis in Papua New Guinea. New England Journal of Medicine. 2002;347(23):1841-8.
7. Krentel A, Fischer P, Manoempil P, Supali T, Servais G, Rückert P. Using knowledge, attitudes and practice [KAP] surveys on lymphatic filariasis to prepare a health promotion campaign for mass drug administration in Alor District, Indonesia. Tropical Medicine & International Health. 2006;11(11):1731-40.
8. Cantey PT, Rao G, Rout J, Fox LM. Predictors of compliance with a mass drug administration programme for lymphatic filariasis in Orissa State, India 2008. Tropical Medicine and International Health. 2010;15(2):224-31. doi: http://dx.doi.org/10.1111/j.1365-3156.2009.02443.x. PubMed PMID: 2010046751.
9. Mathieu E, Lammie PJ, Radday J, Beach MJ, Streit T, Wendt J, et al. Factors associated with participation in a campaign of mass treatment against lymphatic filariasis, in Leogane, Haiti. Annals of Tropical Medicine and Parasitology. 2004;98(7):703-14. doi: 10.1179/000349804x3135. PubMed PMID: WOS:000224651200006.
10. Krentel A, Aunger R. Causal chain mapping: a novel method to analyse treatment compliance decisions relating to lymphatic filariasis elimination in Alor,

Indonesia. Health Policy Plan. 2011. Epub 2011/06/30. doi: 10.1093/heapol/czr048. PubMed PMID: 21712348.
11. Fraser M, Taleo G, Taleo F, Yaviong J, Amos M, Babu M, et al. Evaluation of the program to eliminate lymphatic filariasis in Vanuatu following two years of mass drug administration implementation: results and methodologic approach. The American journal of tropical medicine and hygiene. 2005;73(4):753-8.
12. Nandha B, Sadanandane C, Jambulingam P, Das P. Delivery strategy of mass annual single dose DEC administration to eliminate lymphatic filariasis in the urban areas of Pondicherry, South India: 5 years of experience. Filaria J. 2007;6:7. Epub 2007/08/28. doi: 10.1186/1475-2883-6-7. PubMed PMID: 17718908; PubMed Central PMCID: PMC2020462.
13. Worrell C, Mathieu E. Drug Coverage Surveys for Neglected Tropical Diseases: 10 Years of Field Experience. American Journal of Tropical Medicine and Hygiene. 2012;87(2):216-22.
14. Witt C, Ottesen EA. Lymphatic filariasis: an infection of childhood. Tropical Medicine & International Health. 2001;6(8):582-606.

Appendix 1 Information sheet and informed consent for community survey

(to be read to each respondent)

Good morning / afternoon, my name is _________. I am working with ______. We are doing a survey about the recent special distribution of LF drugs in your community. Your opinion is important to improve the health of the people living in this community and we thank you for your time.

As part of the “Community Based Safety Study of 2-drug versus 3-drug Therapy for Lymphatic Filariasis” that happened in your area recently, we are asking some people who participated in that study to take part in a short survey so that we can understand more about lymphatic filariasis [LOCAL NAME], the drugs used in the distribution and health in general. Your name was selected randomly from the list of people who participated in that special distribution.

It is important that you understand why we are doing this survey, so please read this information sheet or listen carefully as I read it. If you have any more questions, please ask me (the interviewer) and I will try to answer them for you.

We are interested in the experiences people had participating in the special distribution for LF and what people in your community understand about lymphatic filariasis [LOCAL NAME]. We would like to talk to about 400 people in this area so that we can understand better how people felt about taking the LF drugs. Your participation is entirely voluntary and you are under no obligation to participate. Whether or not you choose to participate, your status and access to health care will not be affected in any way. There are no anticipated risks or benefits for you if you choose to participate in this survey.

If you do choose to help with this study, we will only need about 20 minutes of your time to ask you some questions. At any time during this discussion, you are free to stop and withdraw from the study. You do not have to give me (the interviewer) a reason.

During the interview, I will be writing down your responses on this paper. We will not record your name during our discussion today and you will be assigned an identification number for our records. The information that you provide during our discussion will be kept completely confidential. All files will remain with the main investigator and will be kept in a locked cabinet and all electronic files will be password protected. The main research team in this country from _______(Institute name) will access the study files as will researchers from Washington University in St. Louis and the Bruyère Research Institute in Canada, and their ethics boards.

I have read the information sheet provided or it has been read to me concerning this study and I understand what will be required of me if I participate in this study, which will be a verbal interview and discussion.

My questions regarding this study have been answered by: _________________.

I understand that at any time I may withdraw from this study without giving a reason and without having any effect on my access to health care.

I agree to take part in this study.

Signature of the respondent: _______________________________________

Signature of parent/ guardian if respondent under 18 years: ______________________________

Signature of a witness: ____

Signature of the enumerator to indicate that the informed consent has been read and the information sheet given to the respondent: ___________________________________

Appendix 2 Information sheet and informed consent for in depth interviews with key informant

As part of the “Community Based Safety Study of 2-drug versus 3-drug Therapy for Lymphatic Filariasis” that just happened in your area last month, we are asking some people who participated in that study to take part in a verbal discussion so that we can understand more about lymphatic filariasis [or local name], the drugs used in the safety trial and health in general. It is important that you understand why we are doing this survey, so please read this information sheet carefully. If you have any more questions, ask the interviewer and they will try to answer them for you.

We are interested in the experiences people had participating in the safety trial and what they understand about lymphatic filariasis [or local name]. We would like to talk to about 8 people in this area so that we can understand better how people felt about taking the LF drugs. Your participation is entirely voluntary and you are under no obligation to participate. Whether or not you choose to participate, your status and access to health care will not be affected in any way. There are no anticipated risks or benefits to participation in this study.

If you do choose to help with this study, we will only need about one hour of your time to ask you some questions and to discuss informally. At any time during this discussion, you are free to stop and withdraw from the study. You may refuse to answer any of the questions. You do not have to give the interviewer a reason.

The information that you provide during our discussion will be completely confidential and we will not even write down your name or address. We will take some written notes during our discussion and if you agree, we may also audio record the interview using a digital recorder so that it will be easier to remember what we discussed. All digital files will remain with the main investigator and your name and address will not be recorded. We will write down the conversation and store it safely, with a password. Other researchers may ask to look at our discussion together, and we may share it with them, provided that they respect the same rules of confidentiality. Study files may also be accessed by researchers at _________ [local institution], Washington University in St. Louis and the Bruyere Research Institute in Canada and their ethics boards.

I have read the information sheet provided or it has been read to me concerning this study and I understand what will be required of me if I participate in this study, which will be a verbal interview and discussion.

My questions regarding this study have been answered by: __________________.

I understand that at any time I may withdraw from this study without giving a reason and without having any effect on my access to health care.

I agree to take part in this study.

Signature of the respondent: ____

Signature of a witness: ____

Signature of the enumerator to indicate that the informed consent has been read and the information sheet given to the respondent: ___________________________________

Appendix 3 Information sheet and informed consent for focus group discussion participants

As part of the “Community Based Safety Study of 2-drug versus 3-drug Therapy for Lymphatic Filariasis” that just happened in your area, we are asking some people who participated in that study to take part in a focus group discussion so that we can understand more about lymphatic filariasis [or local name], the drugs used in the safety trial and health in general. It is important that you understand why we are doing this survey, so please read this information sheet carefully. If you have any more questions, ask the interviewer and they will try to answer them for you.

We are interested in the experiences people had participating in the safety trial and what they understand about lymphatic filariasis [or local name]. We would like to talk to about 4 groups of people in this area so that we can understand better how people felt about taking the LF drugs. Your participation is entirely voluntary and you are under no obligation to participate. Whether or not you choose to participate, your status and access to health care will not be affected in any way. There are no anticipated benefits to participation in this study.

If you do choose to help with this study, we will only need about one hour of your time to ask you some questions and to discuss informally. At any time during this discussion, you are free to stop and withdraw from the study. You may also refuse to answer any questions. You do not have to give the interviewer a reason.

We request that participants do not share what is discussed during the focus group with others; however there is the risk that confidentiality will not be maintained. The interviewers will maintain confidentiality in the records by not recording the names of the focus group participants. We will take some written notes during our discussion and if you agree, we may also audio record the interview using a digital recorder so that it will be easier to remember what we discussed. All digital files will remain with the main investigator and your name and address will not be recorded. We will write down the conversation and store it safely, with a password. Other researchers may ask to look at our discussion together, and we may share it with them, provided that they respect the same rules of confidentiality. Study files may also be accessed by researchers at [local research institution], Washington University at St. Louis and the Bruyere Research Institute in Canada and their ethics boards.

I have read the information sheet provided or it has been read to me concerning this study and I understand what will be required of me if I participate in this study, which will be a verbal interview and group discussion.

My questions regarding this study have been answered by: _________________.

I understand that at any time I may withdraw from this study without giving a reason and without having any effect on my access to health care.

I agree to take part in this study.

Signature of the respondent: ______________________________________

Signature of a witness: ____

Signature of the enumerator to indicate that the informed consent has been read and the information sheet given to the respondent: ___________________________________

Appendix 4 Community questionnaire

ACCEPTABILITY SURVEY FOR COMMUNITY RESPONDENTS FOLLOWING THE SPECIAL DISTRIBUTION OF LF PILLS

READ OUT LOUD TO RESPONDENT: Thank you for agreeing to be part of this survey today. As we reviewed in the consent form, we are here to gather some information from community members about the special distribution of LF drugs that happened recently in your community on [GIVE DATE].

QNO	Question	Answer and Code	Skip
1.	Before we start, how would you describe your health today?<br>1 2 3 4 5<br>Poor Neutral Excellent		
2.	Within the last year, are you taking medications for any other illnesses (not related to LF)?	a. Yes<br>b. No<br>c. Don't know	
KNOWLEDGE / PERCEPTION OF RISK QUESTIONS REGARDING LF<br>*[Use local names for LF]*			
3.	How would you describe your understanding of LF?<br>1 2 3 4 5<br>No knowledge Average Very good		
		a. Don't know	
4.	How do you think LF is transmitted or moves from person to person?<br><br>**[DON'T READ ALL ANSWERS AND MARK ALL THAT APPLY]**	a. Worms<br>b. Mosquitoes<br>c. Water<br>d. Curse<br>e. Hereditary<br>f. Other<br>g. Don't know	
5.	In your opinion, what is the primary cause of LF?<br><br>**[DON'T READ ALL ANSWERS AND MARK ALL THAT APPLY]**	a. Worms<br>b. Mosquitoes<br>c. Water<br>d. Curse<br>e. Hereditary	

QNO	Question	Answer and Code	Skip
		f. Other	
		g. Don't Know	
6.	Do you think that you can have LF and not be sick?	a. Yes	
		b. Maybe	
		c. No	
		d. Don't Know	
7.	How many people in your village do you think have LF?<br>1 None — 2 — 3 Some — 4 — 5 Many		
		a. Don't know	
8.	Are you concerned about LF personally?<br>1 No, not at all — 2 — 3 Maybe — 4 — 5 Yes, definitely		
		a. Don't know	
PARTICIPATION IN THE SPECIAL DISTRIBUTION OF LF DRUGS			
[READ OUT LOUD TO PARTICIPANT] *Within the last four months, there was a special distribution of LF treatment to some households in your area. I would like to ask you some questions about that special distribution of LF treatment. Our records indicate that you received treatment at that time.*			
9.	Do you remember the special distribution of LF treatment I am referring to?<br>**[IF NO STOP INTERVIEW]**	a. Yes	
		b. No	
10.	Did you swallow all of the drugs you were offered during that distribution?	a. Yes	
		b. No	
11.	How would you describe the information you received about the LF treatment before the pills were given to you:<br>1 — 2 — 3 — 4 — 5		

QNO	Question	Answer and Code	Skip
	Not useful Neutral Very useful		
		a. Don’t know	
TAKING THE LF PILLS DURING THE SPECIAL DISTRIBUTION OF LF DRUGS			
[READ OUT LOUD TO PARTICIPANT] *Let's talk about when you were offered the treatment this year during the special distribution.*			
12.	Can you describe the level of confidence (trust) you had in the drug distribution team?<br>**[READ ALL ANSWERS, ONLY ONE RESPONSE IS POSSIBLE]**	a. A lot of trust	
		b. Neutral	
		c. Not a lot of trust	
		d. Can’t remember / Don’t know	
13.	Did you eat something before or shortly after taking the pills?	a. Yes	
		b. No	
		c. Can’t remember	
14.	Can you describe the emotion or feeling that you had when you took the LF pills during the special distribution?<br>**[DO NOT READ RESPONSES; MARK ALL THAT APPLY]**	a. No emotion or feeling	
		b. Happy	
		c. Relieved	
		d. Nervous / Anxious	
		e. Afraid	
		f. Angry	
		g. Other	
15.	Were you the only member in your household to take the LF pills this time?	a. Yes	
		b. No	
		c. Don’t know	
PERCEPTIONS ABOUT THE PILLS			
16.	How would you describe the taste of the pills that you received?<br>**[READ ALL ANSWERS, ONLY ONE RESPONSE IS POSSIBLE]**	a. Good taste	
		b. No taste	
		c. Bad taste	
		d. Don’t know / No opinion	

QNO	Question	Answer and Code	Skip
17.	A. Here are some faces expressing various feelings. Which face comes closest to how you felt about the number of pills you received?<br>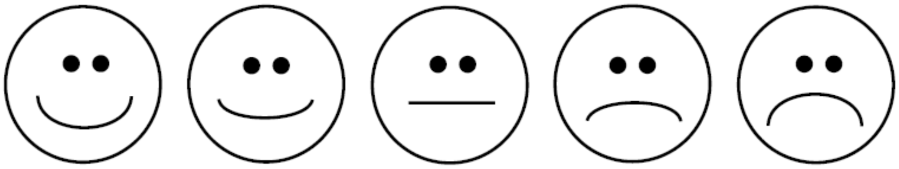		
	B. How do you feel about the number of the pills that you received?<br>1 Not a lot of pills — 2 — 3 Okay — 4 — 5 Many pills		
		a. Don’t know	
SIDE EFFECTS AFTER TAKING THE LF PILLS			
[READ OUT LOUD TO PARTICIPANT] *Let's talk about how your body felt after swallowing the pills during the special LF distribution*			
18.	A. In the day after you took the LF treatment, how did your body feel?<br><br>**[DO NOT READ RESPONSES; MARK ALL THAT APPLY]**	a. Feel the same / didn’t feel anything	
		b. Felt better	
		c. Nauseous	
		d. Headache	
		e. Dizziness	
		f. Fever	
		g. Sleepy	
		h. Stomach pain	
		i. Body pain (muscle / joint)	
		j. Swelling (scrotal)	
		k. Swelling (non-scrotal)	
		l. Vomiting	
		m. Diarrhea	
		n. Itchy skin	
		o. Other	

QNO	Question	Answer and Code	Skip
	B. Here are some faces expressing various feelings. Which face comes closest to how your body felt the day after you took the treatment? 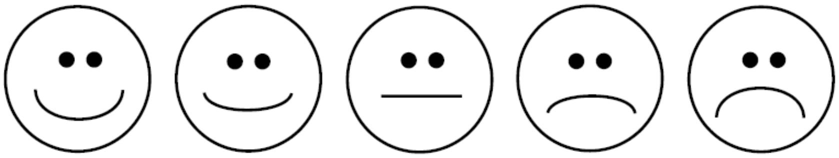		
19.	After you took the LF pills, did any worms come out when you passed stool / defecated / went to the toilet/latrine?	a. Yes	
		b. No	
20.	When you think about how your body felt, which of the following statements are true: **[READ ALL ANSWERS <u>BEFORE</u> RESPONDENT ANSWERS. ONLY ONE RESPONSE IS POSSIBLE]**	a. I had no side effects from the treatment	→Q23
		b. My side effects were mild. They did not interfere with my normal daily activities such as work or school.	
		c. My side effects were moderate. They disrupted my daily activities.	
		d. My side effects were severe. I was confined to bed for at least 1 day.	
		e. My side effects were very severe. I had to go to the hospital.	
21.	Did you speak to a medical professional (nurse, midwife, doctor or community health worker) about the side effects you had this year?	a. Yes	
		b. No	→Q23
22.	How would you describe the support you received to take care of these side effects? ←→ 1 2 3 4 5 / Very poor Okay Excellent		
		a. Don't know	

QNO	Question	Answer and Code	Skip
23.	A. How many people do you know that experienced side effects within one week after taking the treatment?<br>1 No one / 2 / 3 Some / 4 / 5 A lot of people		
		a. Don't know	
	B. Did anyone in your family have side effects the day after taking the treatment?	a. Yes	
		b. No	
		c. Don't know	
	C. Are you concerned about side effects personally?<br>1 No, not at all / 2 / 3 Maybe / 4 / 5 Yes, definitely		
		a. Don't know	
24.	How would you describe your overall health since you took the LF pills?<br>1 Considerably worse / 2 / 3 No change / 4 / 5 Greatly improved		
		a. Don't know	
		**If #3, no change**	→Q26
25.	Do you think this **[Refer to #24]** is related to the pills you took during the special distribution?	a. Yes	
		b. No	
26.	It has been some time now since you took the LF pills during the special distribution. Since then, have you experienced a change in any of the following:		

	Worse	No change	Improved	Not applicable	
a. Itching on my skin					
b. Itching on my head					
c. Feeling in my stomach					

QNO	Question	Answer and Code				Skip
	d. Energy level					
	e. Appetite					
	f. Physical strength					
	g. Quality of sleep					
	h. Skin sores					
QUESTIONS ABOUT ACCEPTABILITY						
27.	In your opinion, which is true about the LF drugs?<br>1 2 3 4 5<br>Not important for my health — Neutral — Very important for my health					
		a. Don't know				
28.	In your opinion, the drugs you took are:<br>1 2 3 4 5<br>Very dangerous — Neutral — Very safe					
		a. Don't know				
	Please rate your opinion on the following statements:	Disagree a lot	Disagree	Agree	Agree a lot	
29.	These drugs work against LF					
30.	These drugs work against itching					
31.	These drugs work against intestinal worms					
32.	I would take this treatment again					
33.	I would recommend this treatment to my relatives					
34.	I would be willing to change my family's routine so that we took the treatment again					
35.	I liked this treatment					
36.	This treatment is a good way to help our health problems here					
37.	Overall this treatment will help my community					
Questions about the Program						
38.	Including this year, how many times have you taken pills for LF?	a. Never				
		b. One time				

QNO	Question	Answer and Code	Skip
		c. 2 or more times	
39.	How important is your participation in the MDA program for your community?<br><br>1 Not important at all — 2 — 3 Neutral — 4 — 5 Very important		
		a. Don't know	
40.	Do you think you should take these pills even if you don't feel sick?	a. Yes<br>b. No<br>c. Don't know	
	KNOWLEDGE / PERCEPTION OF RISK QUESTIONS REGARDING SCABIES		
	[READ OUT LOUD TO PARTICIPANT] *In this next section of questions, I would like to ask you a couple of questions about scabies. Use local names for scabies.*		
41.	Do you know what scabies looks like?	a. Yes<br>b. No<br>c. Don't know	
42.	What do you think causes scabies?<br>**[DO NOT READ RESPONSES; MARK ALL THAT APPLY]**	a. Dietary deficiency (not enough nutrition)<br>b. Small insects that you can't see<br>c. Dirt<br>d. Changes in the weather<br>e. Other<br>f. Don't know	
43.	In your opinion, how many people in your village have scabies?<br><br>1 None — 2 — 3 Some — 4 — 5 Many		
		a. Don't know	

QNO	Question	Answer and Code	Skip
44.	Are you concerned about scabies personally? 1 No, not at all; 2; 3 Maybe; 4; 5 Yes, definitely		
		a. Don't know	
45.	Do you think that scabies can be treated?	a. Yes	
		b. No	
		c. Don't know	
SES INDICATORS			
[READ OUT LOUD TO PARTICIPANT] *Before we finish the interview, I'd like to ask you a few questions about yourself and your household.*			
46.	Is your household income the same amount (fixed) every month?	a. Yes	
		b. No	
		c. Don't know	
47.	From where does the primary source of your household income come?	a. Fishing or farming (agriculture)	
		b. Daily laborer	
		c. Small scale enterprise	
		d. Private employment	
		e. Government / civil servant	
		f. Other	
48.	Can you tell me which of the following items you own? **[READ ALL ANSWERS TO RESPONDENTS AND MARK ALL THAT APPLY]**	a. Clock / watch	
		b. Radio	
		c. Refrigerator	
		d. Television	
		e. Cellphone / Mobile phone	
		f. Bed net	
		g. Bicycle	
		h. Motorcycle / scooter	
		i. Car / Jeep	

QNO	Question	Answer and Code	Skip
		j. Generator<br>k. Solar panels<br>l. Electricity from power company / government	
49.	A. In your household, do you have a toilet? If so, what kind of toilet?	a. Flush toilet<br>b. Pit toilet / latrine<br>c. Manual flush toilet / water-seal with pour	
	B. If no, where do you and your family defecate?<br><br>**[DO NOT READ RESPONSES]**	a. Forest / bush<br>b. Beach<br>c. Other	
50.	What is the highest level of education you have completed:<br><br>**[WRITE OUT GRADE LEVEL: ________]**	a. No school at all<br>b. Completed primary school<br>c. Completed middle school (junior high)<br>d. Completed secondary school<br>e. Completed college / university	
51.	What is your age?<br>**[USE YEAR OF BIRTH IF RESPONDENT DOES NOT KNOW AGE]**		
52.	**[Interviewer to mark without asking]** Primary type of roof:	a. Metal / corrugated iron<br>b. Thatched<br>c. Ceramic tile<br>d. Concrete<br>e. Wood<br>f. Leaf and mud / soil<br>g. Other	
53.	**[Interviewer to mark without asking]** Primary type of floor	a. Tile<br>b. Mud / dirt<br>c. Wood	

QNO	Question	Answer and Code	Skip
		d. Concrete	
		e. Other	
54.	**[Interviewer to mark without asking]** Gender	a. Male	
		b. Female	

Thank you for your time.

[Interviewer to ensure that the participant does not have any further questions or comments about the questionnaire, their participation, etc.]

Appendix 5 Topic guide for In depth Interviews

TOPIC GUIDE – IN DEPTH INTERVIEWS WITH KEY INFORMANTS FOR ACCEPTABILITY OF IDA

MAIN RESEARCH QUESTION: Understanding the feasibility of administering IDA: What are the factors and considerations for the introduction of triple drug therapy for LF elimination into this community?

RATIONALE

Refer to the Information sheet

CONFIDENTIALITY

Refer to the informed consent sheet

WARM-UP QUESTIONS

As you've read the information sheet, you will remember that we are here to collect some information that will help to make your health and the health of people in your village better. So maybe you could tell me a little bit about the health of people in your village...

(If no response... then prompt with: are there many sick people in your village? what do people get sick from in your village?)

LEAD IN TO INTERVIEW

As I mentioned in the beginning of the interview, we are interested in the disease filaria and its treatment. Is that something that you've ever taken medicine for?

If necessary prompt with:

- And what did you take?
- When was that?

KNOWLEDGE ABOUT LF

Can you tell me what you know about the disease LF?

Depending on the identity of the respondent (medical v non-medical) prompt with the following:

- Local names
- How do you get it?
  - Prompt: Would you like to draw here for me how the disease enters the body?
- Tell me what happens to you when you get it.

- Where did you hear about filaria?
- Who do you think gets it most? (Prompt: Men? Women? Children?)
- Do you know anyone who has filaria? Can you tell me about them?
- Is it a problem here?

RECENT SPECIAL FILARIA DRUG DISTRIBUTION

As you know, there was a special distribution of LF drugs trial in your community recently. I would like to talk about that recent drug distribution a bit more now and the future prospects of delivering these drugs again in your community.

Can you tell me about the special LF drug distribution that took place recently?

If necessary prompt with:

- What were people talking about in the community when the special distribution took place?
- What did you understand about this special distribution?

How do you think the distribution of LF pills differs from other health activities in your community? How is it similar?

Can we talk about the people around you and how they felt (emotions) about the treatment given out during the special distribution?

If necessary prompt with:

- Your own family/household: any discussion? Feelings or views about the treatment? Did they take it?
- Neighbours? Feelings or views about the treatment? Did the neighbours take it?
- Community in general? Feelings or views about the treatment? Did they take it?

I'd like us to talk a bit more about the good and bad parts about taking the filaria pills given out during this special distribution.

Let's think about the good things first.

If necessary prompt with:

- What do you think is good about the treatment?
- How did it help you?

- What did you gain from taking the treatment?
- How did your body feel after you took it?

Still thinking about the good things, but now for others, what is good about the treatment for others?

Let's do the same for the bad things, starting with you,

If necessary prompt with:

- What do you think is bad about the treatment?
- How did it hurt you? Did your body feel badly after you took it? Can you explain?

And then for others?

If necessary prompt with:

- Do you know anyone who suffered in some way after taking the treatment?
- How did it hurt them?

Let's talk about the pills themselves a bit. Adults in your community received # [fill in for the specific country] of pills. Can you tell me about that?

If necessary prompt with:

- How did you feel about taking # of pills?
- What did other people in your community say about the # of pills they were asked to take?

What aspects of this special distribution for LF would be most difficult to implement? Easiest?

What suggestions do you have for the messages we might use to promote this special distribution of LF drugs in the community in the future?

If necessary prompt with:

- In your opinion, what's the most important thing about taking the pills?
- What do you think would motivate / encourage people to swallow the pills?
- Who should deliver those messages?

Thinking about your own community, can you think of a group or groups of people who may be hard to distribute the filaria drugs to?

If necessary prompt with:

- Why are they hard to reach?
- Are they hard to reach for other activities as well?
- What advice do you have to reach this group / these groups in future distributions of filarial drugs?

Would you recommend this special drug distribution for LF for your community again next year? Why or why not?

How acceptable do you find this special LF drug distribution is for the health problems in your community?

RESPONDENT ORDERS STATEMENTS:

In this last part of the interview, we will be looking at 5 statements. Let's read them together.

- Take the pills so you won't get filaria.
- Take the pills so your children won't get filaria.
- Take the pills so our community will not get filaria.
- Take the pills so the [district / department name] doesn't get filaria.
- Take the pills so [country name] doesn't get filaria.

Can you put them in order of importance? Why did you arrange them this way?

PLEASE PROBE CONTINUALLY THROUGHOUT WITH THINGS LIKE:

- Why did you say that?
- What do you mean by that?
- Could you tell me a bit more?
- Can you say what you mean by that?
- So then what happened?
- Oh, that makes sense…
- That's interesting…

THANK YOU FOR YOUR TIME!

Make sure they know where to get further information.

Appendix 6 Topic guide for Focus Group Discussions

TOPIC GUIDE
FOCUS GROUP DISCUSSIONS ON THE ACCEPTABILITY OF IDA

MAIN RESEARCH QUESTION
What are the factors and considerations for the introduction of triple drug therapy for LF elimination into this community?

RATIONALE
Refer to the Information sheet

CONFIDENTIALITY
Refer to the informed consent sheet

WARM-UP QUESTIONS
As you've read the information sheet, you will remember that we are here to collect some information that will help the government to make your health and the health of people in your village better. So maybe you could tell me a little bit about the health of people in your village…

(If no response… then prompt with: are there many sick people in your village? what do people get sick from in your village?)

LEAD IN TO DISCUSSION
As I mentioned in the beginning of the interview, we are interested in the disease filaria [use local name] and its treatment. Is that something that you've taken medicine for? And do you remember what you took?

(Interviewer: Oh, that's interesting… maybe we can talk about that a bit more later…)

KNOWLEDGE ABOUT LF

Can you please tell me what you know about the disease LF?

Depending on the identity of the group (medical v non-medical) prompt with the following:

- Local names
- How do you get it?
- Tell me what happens to you when you get it.
- Where did you hear about filaria?
- Who do you think gets it most? (Prompt: Men? Women? Children?)

- Do you know anyone who has filaria? Can you tell me about them?
- Do you think it is a problem here?

Past MDA activities

[FOR FGD with CDDs]

Could you tell me about your role in the distribution for LF drugs in your area?

If necessary prompt with:

- Any part you played in the promotion and/or distribution of the drugs this year or before?
- How did you tell people about the treatment day?
- What was the role of community leaders: village leader, school teachers, church or mosque leaders, neighbours, health staff?

In order to know that people actually take the pills that are given to them, it is we ask that community members take the pills in front of the distributor. I'd like to talk a bit more about that. Can you tell me how this happened in your community?

If necessary prompt with:

- How do you think people feel about taking pills in front of the distributor?
- What recommendations do you have to improve the frequency of people taking pills in front of the distributor?

How do you think the distribution of LF pills differs from other health activities in your community? How is it similar?

Recent special filaria drug distribution

As you know, there was a special distribution of LF tablets in your community recently. I would like to talk about that recent drug distribution a bit more.

Can you tell me about the special LF drug distribution that took place recently?

If necessary prompt with:

- How was it different from past MDA activities?

- What were people talking about in the community when the special distribution took place?

Can we talk about the people around you and how they felt (emotions) about the treatment given out during the special distribution?

If necessary prompt with:

- Your own family/household: any discussion? Feelings or views about the treatment? Did they take it?
- Neighbours? Feelings or views about the treatment? Did the neighbours take it?
- Community in general? Feelings or views about the treatment? Did they take it?

I'd like us to talk a bit more about the good and bad parts about taking the filaria pills given out during this special distribution.

Let's think about the good things first.

If necessary prompt with:

- What do you think is good about the treatment?
- How does it help?
- What did you gain from taking the treatment?
- How did your body feel after you took it?

Still thinking about the good things, but now for others, what is good about the treatment for others?

Let's do the same for the bad things, starting with you,

If necessary prompt with:

- What do you think is bad about the treatment?
- How did it hurt you? Did your body feel badly after you took it? Can you explain?

And then for others?

If necessary prompt with:

- Do you know anyone who suffered in some way after taking the treatment?
- How did it hurt them?

Let's talk about the pills themselves a bit. Adults in your community received # [fill in for the specific country] of pills. Can you tell me about that?

If necessary prompt with:

- How did you feel about taking # of pills?
- What did other people in your community say about the # of pills they were asked to take?

What aspects of this special distribution for LF would be most difficult to implement? Easiest?

What suggestions do you have for the messages we might use to promote this special distribution of LF drugs in the community in the future?

If necessary prompt with:

- In your opinion, what's the most important thing about taking the pills?
- What do you think would motivate / encourage people to swallow the pills?
- Who should deliver those messages?

Thinking about your own community, can you think of a group or groups of people who may be hard to distribute the filaria drugs to?

If necessary prompt with:

- Why are they hard to reach?
- Are they hard to reach for other activities as well?
- What advice do you have to reach this group / these groups in future distributions of filarial drugs?

Would you recommend this special drug distribution for LF for your community again next year? Why or why not?

[FOR FGD with CDDs]

If this special distribution occurred again next year here, what would you need to help you to distribute it?

If necessary prompt with:

- Specific tools (materials)?

- Training?
- Can you tell me about any specific challenges that we need to consider?

PLEASE PROBE CONTINUALLY THROUGHOUT WITH THINGS LIKE:

- Why did you say that?
- What do you mean by that?
- Could you tell me a bit more?
- Can you say what you mean by that?
- So then what happened?
- Oh, that makes sense…
- That's interesting…

THANK YOU FOR YOUR TIME! PLEASE MAKE SURE THEY KNOW WHERE TO GET FURTHER INFORMATION.

Appendix 7 Topic Guide for Focus Group Discussion in Treatment Naïve Areas

TOPIC GUIDE
FOCUS GROUP DISCUSSIONS ON THE ACCEPTABILITY OF IDA (IN TREATMENT NAÏVE AREAS)

MAIN RESEARCH QUESTION
What are the factors and considerations for the introduction of triple drug therapy for LF elimination into this community?

RATIONALE
Refer to the Information sheet

CONFIDENTIALITY
Refer to the informed consent sheet

WARM-UP QUESTIONS
As you've read the information sheet, you will remember that we are here to collect some information that will help the government to make your health and the health of people in your village better. So maybe you could tell me a little bit about the health of people in your village…

(If no response… then prompt with: are there many sick people in your village? what do people get sick from in your village?)

LEAD IN TO DISCUSSION
As I mentioned in the beginning of the interview, we are interested in the disease filaria [use local name] and its treatment. Is that something that you've taken medicine for? And do you remember what you took?

(Interviewer: Oh, that's interesting… maybe we can talk about that a bit more later…)

KNOWLEDGE ABOUT LF

Can you please tell me what you know about the disease LF?

Depending on the identity of the group (medical v non-medical) prompt with the following:

- Local names
- How do you get it?
- Tell me what happens to you when you get it.

- Where did you hear about filaria?
- Who do you think gets it most? (Prompt: Men? Women? Children?)
- Do you know anyone who has filaria? Can you tell me about them?
- Do you think it is a problem here?

Recent special filaria drug distribution

As you know, there was a special distribution of LF tablets in your community recently. I would like to talk about that recent drug distribution a bit more.

Can you tell me about the special LF drug distribution that took place recently?

If necessary prompt with:

- Did you have a special role?
- What were people talking about in the community when the special distribution took place?

Can we talk about the people around you and how they felt (emotions) about the treatment given out during the special distribution?

If necessary prompt with:

- Your own family/household: any discussion? Feelings or views about the treatment? Did they take it?
- Neighbours? Feelings or views about the treatment? Did the neighbours take it?
- Community in general? Feelings or views about the treatment? Did they take it?

I'd like us to talk a bit more about the good and bad parts about taking the filaria pills given out during this special distribution.

Let's think about the good things first.

If necessary prompt with:

- What do you think is good about the treatment?
- How does it help?
- What did you gain from taking the treatment?
- How did your body feel after you took it?

Still thinking about the good things, but now for others, what is good about the treatment for others?

Let's do the same for the bad things, starting with you,

If necessary prompt with:

- What do you think is bad about the treatment?
- How did it hurt you? Did your body feel badly after you took it? Can you explain?

And then for others?

If necessary prompt with:

- Do you know anyone who suffered in some way after taking the treatment?
- How did it hurt them?

Let's talk about the pills themselves a bit. Adults in your community received # [fill in for the specific country] of pills. Can you tell me about that?

If necessary prompt with:

- How did you feel about taking # of pills?
- What did other people in your community say about the # of pills they were asked to take?

What aspects of this special distribution for LF would be most difficult to implement? Easiest?

What suggestions do you have for the messages we might use to promote this special distribution of LF drugs in the community in the future?

If necessary prompt with:

- In your opinion, what's the most important thing about taking the pills?
- What do you think would motivate / encourage people to swallow the pills?
- Who should deliver those messages?

Thinking about your own community, can you think of a group or groups of people who may be hard to distribute the filaria drugs to?

If necessary prompt with:

- Why are they hard to reach?

- Are they hard to reach for other activities as well?
- What advice do you have to reach this group / these groups in future distributions of filarial drugs?

Would you recommend this special drug distribution for LF for your community again next year? Why or why not?

[FOR FGD with CDDs]

If this special distribution occurred again next year here, what would you need to help you to distribute it?

If necessary prompt with:

- Specific tools (materials)?
- Training?
- Can you tell me about any specific challenges that we need to consider?

PLEASE PROBE CONTINUALLY THROUGHOUT WITH THINGS LIKE:

- Why did you say that?
- What do you mean by that?
- Could you tell me a bit more?
- Can you say what you mean by that?
- So then what happened?
- Oh, that makes sense…
- That's interesting…

THANK YOU FOR YOUR TIME! PLEASE MAKE SURE THEY KNOW WHERE TO GET FURTHER INFORMATION.
